# SARS-CoV-2 3CLpro mutations T21I and E166A confer differential resistance to simnotrelvir, bofutrelvir, and ensitrelvir

**DOI:** 10.1128/jvi.02223-25

**Published:** 2026-04-27

**Authors:** Lu Chen, Haixia Su, Weijuan Shang, Tianqing Nie, Wenhua Kuang, Chenchen Wang, Qiang Shao, Leike Zhang, Wen Liu, Yechun Xu, Yumin Zhang

**Affiliations:** 1 Key Laboratory of Virology and Biosafety, Wuhan Institute of Virology, Chinese Academy of Sciences 74614 , Wuhan, China; 2 University of Chinese Academy of Sciences 74519, Beijing, China; 3 State Key Laboratory of Drug Research, Shanghai Institute of Materia Medica, Chinese Academy of Sciences 58298 , Shanghai, China; 4 Microbiome Research Group, Research Centre for Life Science and Healthcare, Nottingham Ningbo China Beacons of Excellence Research and Innovation Institute (CBI), University of Nottingham Ningbo China, Ningbo, China; 5 Hubei Jiangxia Laboratory, Wuhan, China; 6 College of Biological and Pharmaceutical Sciences, China Three Gorges University 643319, Yichang, China; 7 School of Pharmaceutical Science and Technology, Hangzhou Institute for Advanced Study, University of Chinese Academy of Sciences 638898, Hangzhou, China; Loyola University Chicago - Health Sciences Campus, Maywood, Illinois, USA

**Keywords:** 3CLpro, drug resistance, E166A, simnotrelvir, bofutrelvir

## Abstract

Inhibiting the catalytic activity of 3CLpro is a mainstream strategy to block coronavirus replication. However, the appearance of SARS-CoV-2 3CLpro resistance to protease inhibitors raises concerns for effective therapies. In this work, we first investigated the resistance profile of simnotrelvir, an approved anti-SARS-CoV-2 drug that targets 3CLpro. We found that the T21I/E166A mutations in 3CLpro equally emerged when SARS-CoV-2 was passaged in the HEK293T-hACE2 cells with increasing concentrations of simnotrelvir. The SARS-CoV-2 isolate carrying 3CLpro^T21I/E166A^ (SARS2-T21I/E166A) showed cross-resistance to simnotrelvir, nirmatrelvir, and ensitrelvir, but not significant resistance to bofutrelvir. Biochemical and cellular assays confirmed that 3CLpro^T21I/E166A^ was associated with the differential resistance to these protease inhibitors. Crystallographic structural analysis indicated that the alanine substitution disrupted hydrogen bonding interactions surrounding the γ-lactam rings (P1) of the inhibitors, which is similar to the model rebuilding observed with the previously reported E166V mutation. However, in contrast to the valine substitution, the alanine substitution resulted in a more spacious S2 subsite, thereby causing stronger interaction between the P1 and residues F140 and Ser1 of protomer B. Further computational simulations demonstrated that the covalent binding of bofutrelvir preserves strong binding affinity despite modifications in the S2 subsite caused by the E166A mutation, suggesting that inhibitors containing an aldehyde warhead may partially overcome resistance. Notably, both simnotrelvir and bofutrelvir exhibited therapeutic efficacy against the SARS2-T21I/E166A variant in K18-hACE2 mice. These findings advance our understanding of the resistance profiles and mechanistic underpinnings of SARS-CoV-2 3CLpro and underscore the necessity for diversified antiviral therapeutic strategies.

**IMPORTANCE:**

Considering that the nirmatrelvir-resistant SARS-CoV-2 has emerged in immunocompromised patients who received long-term Paxlovid therapy, it is essential to investigate the response of resistance 3CLpro mutants to various protease inhibitors. Simnotrelvir, a novel inhibitor targeting SARS-CoV-2 3CLpro, has been authorized for the treatment of mild-to-moderate COVID-19 in China and has treated over 1 million patients. However, the resistance profile of simnotrelvir to SARS-CoV-2 remains unknown. Here, we identified that 3CLpro with T21I/E166A mutations confers resistance to simnotrelvir and showed cross-resistance to nirmatrelvir and ensitrelvir, but not bofutrelvir. More importantly, we further revealed that E166A showed a novel resistance mechanism to both the covalent inhibitors consisting of a γ-lactam ring and non-covalent inhibitors like ensitrelvir, which is different from that of E166V previously reported. In contrast, bofutrelvir maintains high affinity to T21I/E166A, suggesting that inhibitors with aldehyde warhead can partly neutralize the resistance.

## INTRODUCTION

Severe acute respiratory syndrome coronavirus 2 (SARS-CoV-2), the pathogen leading to COVID-19, continues to spread around the world. Numerous variants of concern (VOCs) have emerged due to rapid evolution, leading to multiple waves of infection (1). The efficacy of vaccines and therapeutic monoclonal antibodies that target the spike protein of SARS-CoV-2 varies significantly on the basis of circulating VOCs, but protease inhibitors targeting viral 3CL proteases (3CLpro), such as nirmatrelvir (NMT) (PF-07321332), ensitrelvir (EST) (S-217622), and simnotrelvir (SNT), remain active (2–4). These protease inhibitors interfere with 3CLpro via their covalent or non-covalent binding to the catalytic active site, thereby blocking the cleavage of the viral polypeptides pp1a and pp1ab. Although some other viral proteins and host factors have been proven druggable against coronaviruses, 3CLpro is still one of the most attractive and dominant targets for the development of effective and broad-spectrum anti-CoV drugs (5).

Treatment with antivirals can lead to the emergence of resistant viral strains, resulting in therapeutic failure, as observed in clinical cases involving the human immunodeficiency virus (HIV), the hepatitis C virus (HCV), and influenza virus infections (6–8). In recent years, mutations occurred in the 3CLpro gene that confer SARS-CoV-2 resistance to nirmatrelvir have been identified, raising concerns regarding treatment efficacy with the increasing clinical application of 3CLpro inhibitors. Studies of *in vitro* passage in the presence of nirmatrelvir for the selection of 3CLpro mutants or adapted gain-of-function viruses revealed that E166A/V conferred the strongest resistance by reducing susceptibility by 80-fold to 100-fold in cell-based assays and by 1,000-fold in enzymatic assays (9–13). The amino acid substitutes of E166 directly disrupt interactions with nirmatrelvir or alter the conformation of the nirmatrelvir-binding pocket. Structural analyses of the 3CLpro-nirmatrelvir complex have demonstrated that the E166 residue plays a critical role in occupying the S1 subsite and forms four hydrogen bonds (H-bonds) with nirmatrelvir or the substrates, while these H-bonds can be disrupted by valine substitution (14). Although the price of the resistance given by E166A/V is fitness loss, viral fitness can be partially restored through compensatory substitutions such as L50F (S2 subsite) and T21I (S4′ subsite) (9, 13). In addition to the amino acid substitutes selected by inhibitors, some naturally occurring alterations in 3CLpro, including S144A, ΔP168, A173V, and E166D/G, also confer resistance to nirmatrelvir (15–17). In most cases, the nirmatrelvir-resistant E166A/V has significant cross-resistance to other non-covalent and covalent inhibitors, such as GC-376 and ensitrelvir (18, 19). Consequently, it is critical to evaluate and compare the resistance profiles generated by the different 3CLpro inhibitors to enhance our understanding of the common and specific resistance mechanisms, and a comprehensive characterization of authentic drug-resistant SARS-CoV-2 strains will provide valuable insights into mutant virus infections and therapeutic strategies.

In a previous study, we reported the development of a novel inhibitor targeting SARS-CoV-2 3CLpro, simnotrelvir, with an enthalpy-driven binding model in the active pocket (4). Simnotrelvir was optimized from boceprevir, an approved protease inhibitor for HCV, and simnotrelvir covalently inhibits SARS-CoV-2 3CLpro with high selectivity. It has been authorized for the treatment of mild-to-moderate COVID-19 in China, demonstrating clinical benefits by shortening the resolution time of symptoms among adult patients (20). Given the nirmatrelvir resistance and potential cross-resistance mentioned above, further investigations into the resistant spectrum of other 3CLpro inhibitors should be conducted. More importantly, considering that nirmatrelvir-resistant SARS-CoV-2 has emerged in immunocompromised patients who have received long-term Paxlovid therapy (21), characterizing resistant SARS-CoV-2 *in vitro* and *in vivo*, and investigating the response of resistant 3CLpro mutants and the corresponding resistant viruses to various protease inhibitors are essential.

In this study, we compare the mutation profiles of SARS-CoV-2 in response to simnotrelvir and nirmatrelvir and find that T21I and E166A are the common substitutes in 3CLpro. Then, we indicate that the SARS-CoV-2 isolate bearing 3CLpro^T21I/E166A^ is cross-resistant to simnotrelvir and ensitrelvir, but not significantly resistant to bofutrelvir (FB2001) in cell assays. Systematic methodologies elucidate the mechanisms of the different resistant levels of 3CLpro^T21I/E166A^ to these protease inhibitors. Our findings contribute to the knowledge of 3CLpro resistance from virological, biochemical, and molecular perspectives and offer insights into the development of additional antivirals capable of overcoming the cross-resistance of SARS-CoV-2 with diverse mechanisms of action.

## RESULTS

### T21I and E166A are the common substitutions selected by simnotrelvir and nirmatrelvir

**Fig 1:**
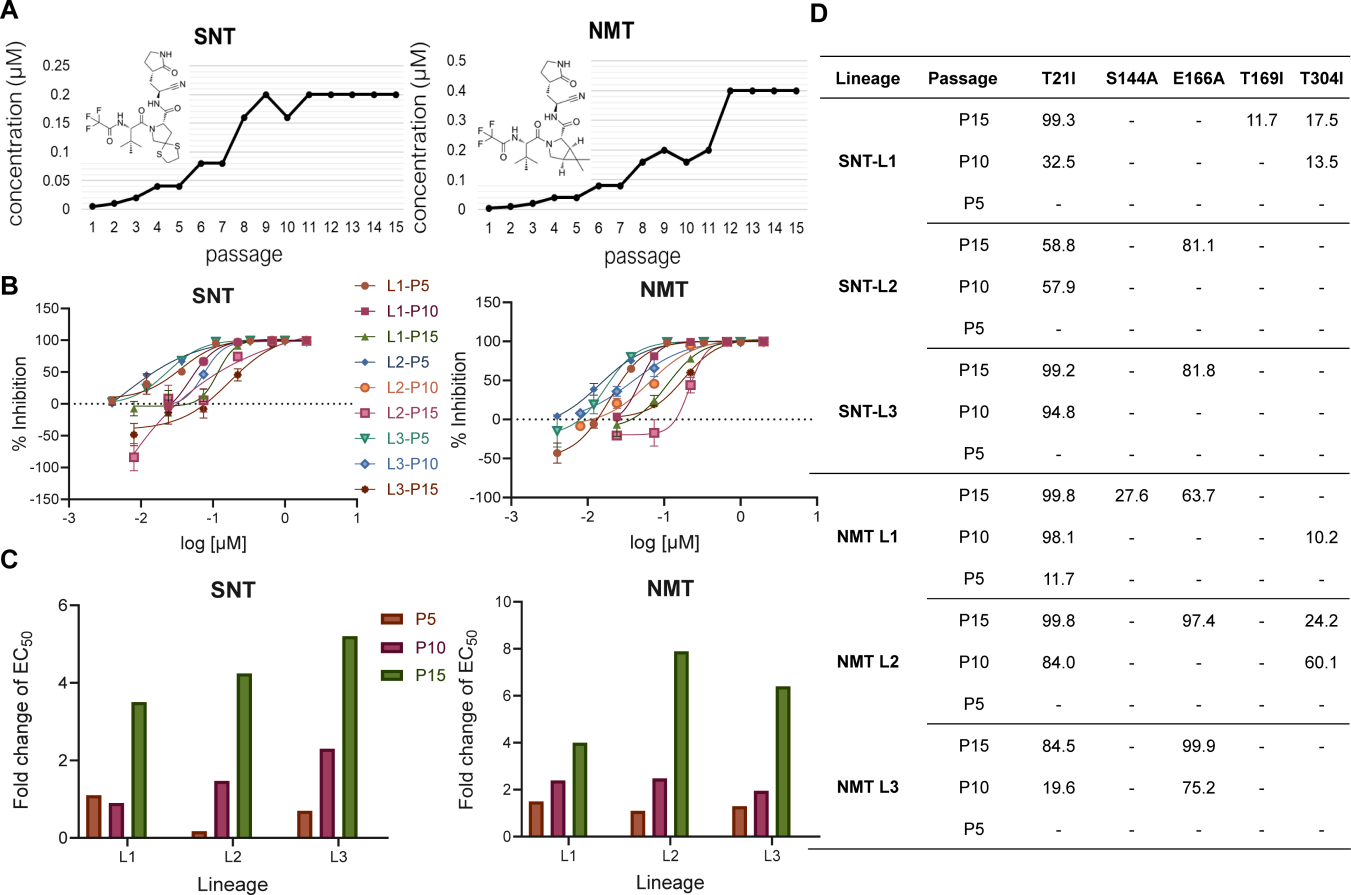
Identification of SNT and NMT resistance of SARS-CoV-2 in HEK293T-hACE2 cells. (**A**) The scheme of the cell-based passaging experiments for SARS-CoV-2 mutant selection in HEK293T-hACE2 cells. (**B**) Changes in drug response during serial passages of SARS-CoV-2 with simnotrelvir and nirmatrelvir. HEK293T-hACE2 cells were infected in triplicate (L1–L3) with SARS-CoV-2 and passaged to fresh cells every 3 days for 10 or 15 passages. Validation of simnotrelvir and nirmatrelvir resistance for the indicated passages from each of the three lineages. (**C**) The fold changes of EC_50_ values of viral passages from each lineage by nirmatrelvir and simnotrelvir compared to the EC_50_ values of SARS2-WT. The bars represent one of three independent experiments. (**D**) Mutations in 3CLpro found in the indicated passages from each lineage.

To explore and compare the resistance of SARS-CoV-2 to nirmatrelvir and simnotrelvir, the SARS-CoV-2 delta variant (B.1.617.2, IVCAS6.7585) was subjected to serial passaging in the presence of progressively increasing concentrations of each protease inhibitor in the HEK293T-hACE2 cells (Fig. 1A). The initial viral generation was established in triplicate to represent three independent lineages, which were passaged accordingly. After 15 passages, all three lineages exhibited a significant degree of resistance to the respective inhibitors (Fig. 1B), with the half maximum effective concentration (EC_50_) values increasing 5-fold to 8-fold compared with those of the original wild-type virus (Fig. 1C). To identify the mutations associated with resistance, we performed whole-genome sequencing of the virus populations collected at passages 5, 10, and 15 for each lineage using next-generation sequencing (NGS). Given that the inhibitors under investigation exert antiviral effects by targeting 3CLpro, we focused primarily on the amino acid modifications occurring within the 3CLpro gene. The findings revealed the presence of T21I, S144A, E166A, T169I, and T304I substitutions in 3CLpro, particularly the accumulation of the E166A substitution along with T21I in the virus populations that passed in all three nirmatrelvir lineages and two simnotrelvir lineages (Fig. 1D). In addition to 3CLpro mutations, mutations in other genome regions were also detected during the stepwise passaging process (Table S1). These results indicate that SARS-CoV-2 developed 3CLpro resistance profiles similar to those of simnotrelvir, as observed with nirmatrelvir resistance, and the sequential emerging substitutions of T21I and E166A are the common features potentially associated with nirmatrelvir and simnotrelvir resistance.

### The SARS-CoV-2 isolate bearing 3CLpro^T21I/E166A^ is cross-resistant to simnotrelvir, nirmatrelvir, and ensitrelvir, but not significant resistance to bofutrelvir in cells

**Fig 2:**
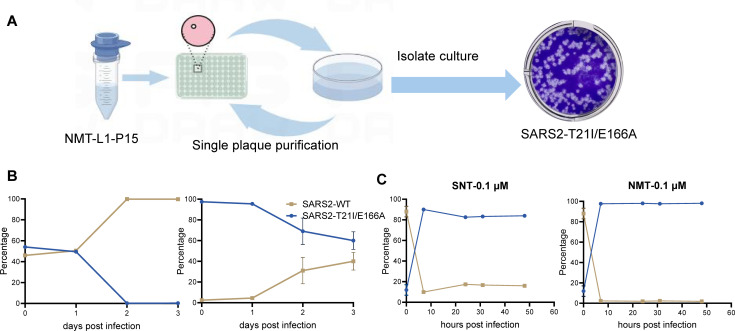
Isolation and characterization of SARS2-T21I/E166A. (**A**) The scheme of single plaque isolation, 5–10 single plaques were separated and selected by limiting dilution of nirmatrelvir lineage 1 of passage 15. Every single plaque was propagated in a 10-mm dish and then the limiting dilution and single plaque selection was repeated for another two rounds. Sanger sequencing for the 3CLpro gene of each plaque to confirm T21I/E166A mutations and set one isolate (SARS2-T21I/E166A) as representative. (**B, C**) Growth competition experiments between the SARS2-T21I/E166A and SARS2-WT strains in the HEK293T-hACE2 cells without (**B**) or with (C) drugs. SARS2-T21I/E166A and SARS2-WT were mixed at different ratios at the beginning in the culture medium containing simnotrelvir or nirmatrelvir, and the frequency of each virus was determined via a next-generation sequencing (NGS) analysis, with the specific 3CLpro mutations used as viral markers. SNT, simnotrelvir; NMT, nirmatrelvir.

We isolated single plaques from simnotrelvir lineage 1 at passage 15 (SNT-L1-P15) to investigate the growth and drug resistance characteristics of the authentic SARS-CoV-2 isolate, which possesses the T21I and E166A in 3CLpro. Following three rounds of plaque isolation and purification, we successfully obtained a purified SARS-CoV-2 strain bearing 3CLpro^T21I/E166A^ (SARS2-T21I/E166A) (Fig. 2A). To identify potential mutations associated with drug resistance, we sequenced the whole genome of SARS2-T21I/E166A through NGS. The genome sequence revealed that SARS2-T21I/E166A has mutations in nonstructural proteins 3, 5, and 14, and the 3′-noncoding regions, and contains T21I and E166A mutations within 3CLpro (Table S2). Furthermore, we observed that SARS2-T21I/E166A remained genetically stable, as no additional substitutions were detected after five viral passages in cells, without any inhibitor selection (data not shown).

We subsequently assessed the sensitivity of SARS2-T21I/E166A to simnotrelvir and nirmatrelvir in the Vero E6 and HEK293T-hACE2 cells, respectively. The results indicated that SARS2-T21I/E166A was substantially resistant to simnotrelvir and nirmatrelvir, with increased EC_50_ values of 8.6-fold to 9.2-fold, which are 4.3-fold to 5.4-fold greater than those of the SARS-CoV-2 wild-type (SARS2-WT), respectively (Table 1). These findings suggest that SARS2-T21I/E166A has cross-resistance to simnotrelvir and nirmatrelvir. Direct competition experiments conducted in HEK293T-hACE2 cells revealed that the wild-type strain had greater fitness in the absence of inhibitors but had inferior growth than SARS2-T21I/E166A in both simnotrelvir- and nirmatrelvir-containing medium (Fig. 2B and C), indicating that SARS2-T21I/E166A has a competitive advantage over SARS2-WT in terms of simnotrelvir or nirmatrelvir stress *in vitro*.

**TABLE 1:** Comparison of the drug susceptibility of SARS2-WT and SARS2-T21I/E166A to simnotrelvir, nirmatrelvir, bofutrelvir, and ensitrelvir in a cell-based assay^*a*^

Inhibitor	SARS-CoV-2 strain	Vero E6		HEK-293T-hACE2	
		EC_50_ (μM)	Fold to WT	EC_50_ (nM)	Fold to WT
SNT	SARS2-WT	2.45 ± 1.15	5.4	91.4 ± 6.39	4.3
	SARS-T21I/E166A	13.3 ± 1.47		392 ± 17.5	
NMT	SARS2-WT	0.594 ± 0.0827	9.2	8.96 ± 2.28	8.6
	SARS-T21I/E166A	5.45 ± 1.43		77.4 ± 1.26	
BFT	SARS2-WT	0.185 ± 0.0202	2.4	4.26 ± 0.240	<1.0
	SARS-T21I/E166A	0.447 ± 0.150		1.76 ± 0.311	
EST	SARS2-WT	0.122 ± 0.0514	8.8	6.58 ± 1.83	49
	SARS-T21I/E166A	1.08 ± 0.120		323 ± 25.6	

^*a*^ Each EC_50_ value represents one of three independent experiments. The values in bold indicate the EC_50_ fold changes of SARS2-T21I/E166A relative to the EC_50_ against SARS2-WT. SNT, simnotrelvir; NMT, nirmatrelvir; BFT, bofutrelvir; EST, ensitrelvir.

We subsequently intended to know whether the SARS2-T21I/E166A evolved from simnotrelvir pressure is resistant to other protease inhibitors, especially in which bofutrelvir and ensitrelvir, which have not been sufficiently studied in drug resistance. The results of the cell-based assay indicated that SARS2-T21I/E166A was substantially resistant to ensitrelvir, with EC_50_ values increased by 49-fold and 8.8-fold compared with those of SARS2-WT in the HEK293T-hACE2 and Vero E6 cells, respectively. Interestingly, bofutrelvir still exhibited effective inhibitory activity against SARS2-T21I/E166A, with EC_50_ values ranging from 1.76 to 447 nM in the two cell lines.

### Validating 3CLpro^T21I/E166A^ resistance to the inhibitors via biochemical study

**Fig 3:**
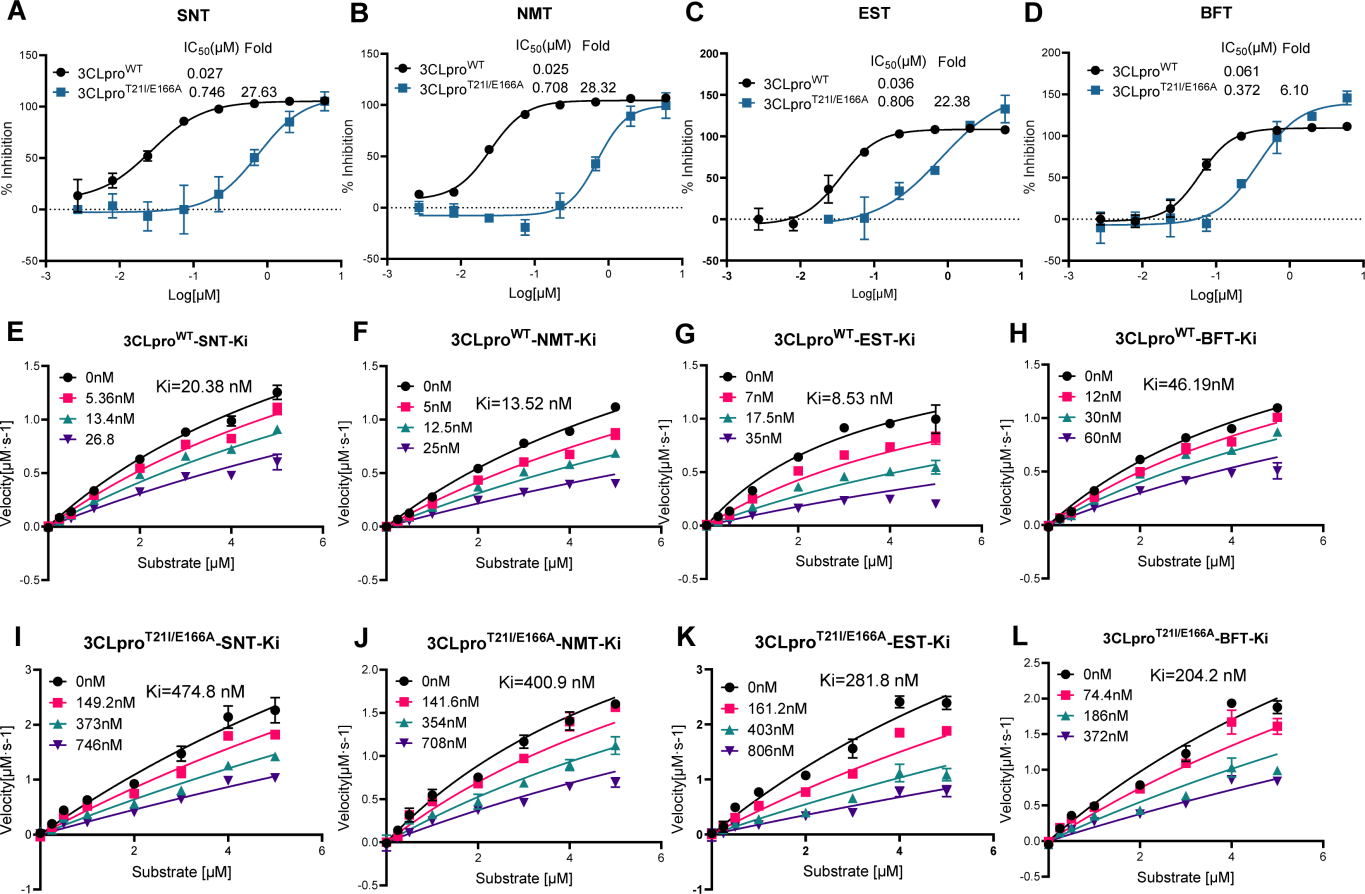
The biochemical response of 3CLpro^WT^ and 3CLpro^T21I/E166A^ to simnotrelvir, nirmatrelvir, ensitrelvir, and bofutrelvir via a FRET-based enzymatic assay. (**A–D**) The inhibitory activities of simnotrelvir, nirmatrelvir, ensitrelvir, and bofutrelvir against SARS-CoV-2 3CLpro^WT^ and 3CLpro^T21I/E166A^ were evaluated, and the data represent the means ± SDs of three independent measurements. (**E–L**) Ki values of 3CLpro^WT^ and 3CLpro^T21I/E166A^ to simnotrelvir, nirmatrelvir, ensitrelvir, and bofutrelvir calculated by their velocities with the Morison equation. SNT, simnotrelvir; NMT, nirmatrelvir; EST, ensitrelvir; BFT, bofutrelvir.

To validate the role of the substitutions in 3CLpro in decreasing the susceptibility of SARS2-T21I/E166A to simnotrelvir, nirmatrelvir, ensitrelvir, and bofutrelvir, we evaluated the susceptibility of recombinant 3CLpro^T21I/E166A^ to these protease inhibitors using a FRET-based enzymatic assay. The IC_50_ values for 3CLpro^T21I/E166A^ for nirmatrelvir, simnotrelvir, and ensitrelvir markedly increased by 27.63-fold, 28.32-fold, and 22.38-fold, respectively, compared with those of the recombinant wild-type 3CLpro (3CLpro^WT^) (Fig. 3A through C). In contrast, the IC_50_ value for 3CLpro^T21I/E166A^ against bofutrelvir was 372 nM, representing only a 6.10-fold increase relative to that of 3CLpro-WT (Fig. 3D). The Ki values of 3CLpro^T21I/E166A^ for simnotrelvir, nirmatrelvir, ensitrelvir, and bofutrelvir were 474.8, 400.9, 281.8, and 204.2 nM, respectively, and their corresponding values of 3CLpro-WT were 20.38, 13.52, 8.53, and 46.19 nM (Fig. 3E through L). Kinetic studies of the initial velocity revealed that the catalytic efficacy of 3CLpro^T21I/E166A^ was significantly diminished (3CLpro^T21I/E166A^ Kcat/Km = 0.638 s^−1^·µM^−1^ vs. 3CLpro^WT^ Kcat/Km = 1.50 s^−1^·µM^−1^). These results indicate that T21I and E166A confer substantial resistance to simnotrelvir, nirmatrelvir, and on 3CLpro, whereas the substitutes can impair the enzymatic activity. Notably, SARS-CoV-2 Omicron 3CLpro^T21I/E166A^ (with additional P132H) also significantly reduced susceptibility to nirmatrelvir and simnotrelvir (Fig. S1). Thermodynamic stability analysis revealed that 3CLpro^T21I/E166A^ presented a lower degree of protein stabilization than WT when co-incubated with simnotrelvir, nirmatrelvir, ensitrelvir, or bofutrelvir, as a decreased ΔTm was observed, although the ΔTm of the 3CLpro^T21I/E166A^-bofutrelvir complex was much higher than those of the 3CLpro^T21I/E166A^ complexed with the other protease inhibitors (Fig. S2). These findings demonstrate that T21I and E166A contribute to 3CLpro resistance to simnotrelvir, ensitrelvir, and bofutrelvir by reducing inhibitor affinity in the catalytic site, and the resistance to bofutrelvir was comparably attenuated.

### The binding modes of simnotrelvir, bofutrelvir, and ensitrelvir with 3CLpro^T21I/E166A^

**Fig 4:**
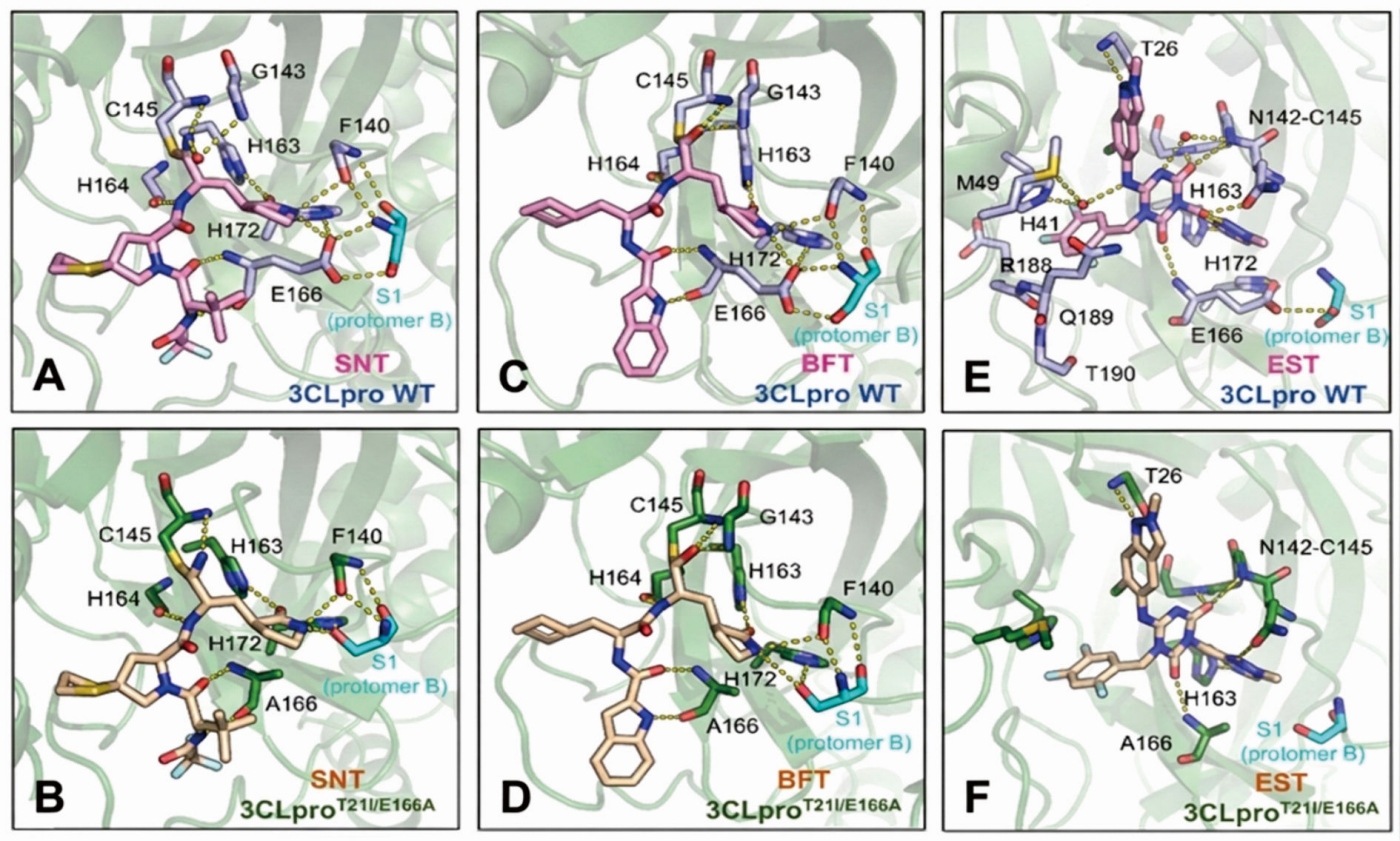
Comparison of the binding modes of SNT, BFT, and EST with SARS-CoV-2 3CLpro T21I/E166A mutant and the wild-type (WT). Only catalytic pockets of 3CLpro are present in the landscapes. (**A and B**) Interactions between SNT and WT (**A**) and T21I/E166A (**B**). (**C and D**) Interactions between BFT and WT (**C**) and T21I/E166A (**D**). (**E and F**) Interactions between EST and WT (**E**) and T21I/E166A (**F**). The WT protein is displayed in a slate-colored cartoon, with the bound small molecules SNT, BFT, and EST shown as pink sticks. The T21I/E166A protein is displayed in a deep green cartoon, with the bound small molecules SNT, BFT, and EST shown as wheat-colored sticks. H-bonds are represented as yellow-dashed lines. The crystal structures of the WT complexed with SNT, BFT, and EST were extracted from PDB under the accession codes 8IGX, 6LZE, and 8HBK, respectively.

**Fig 5:**
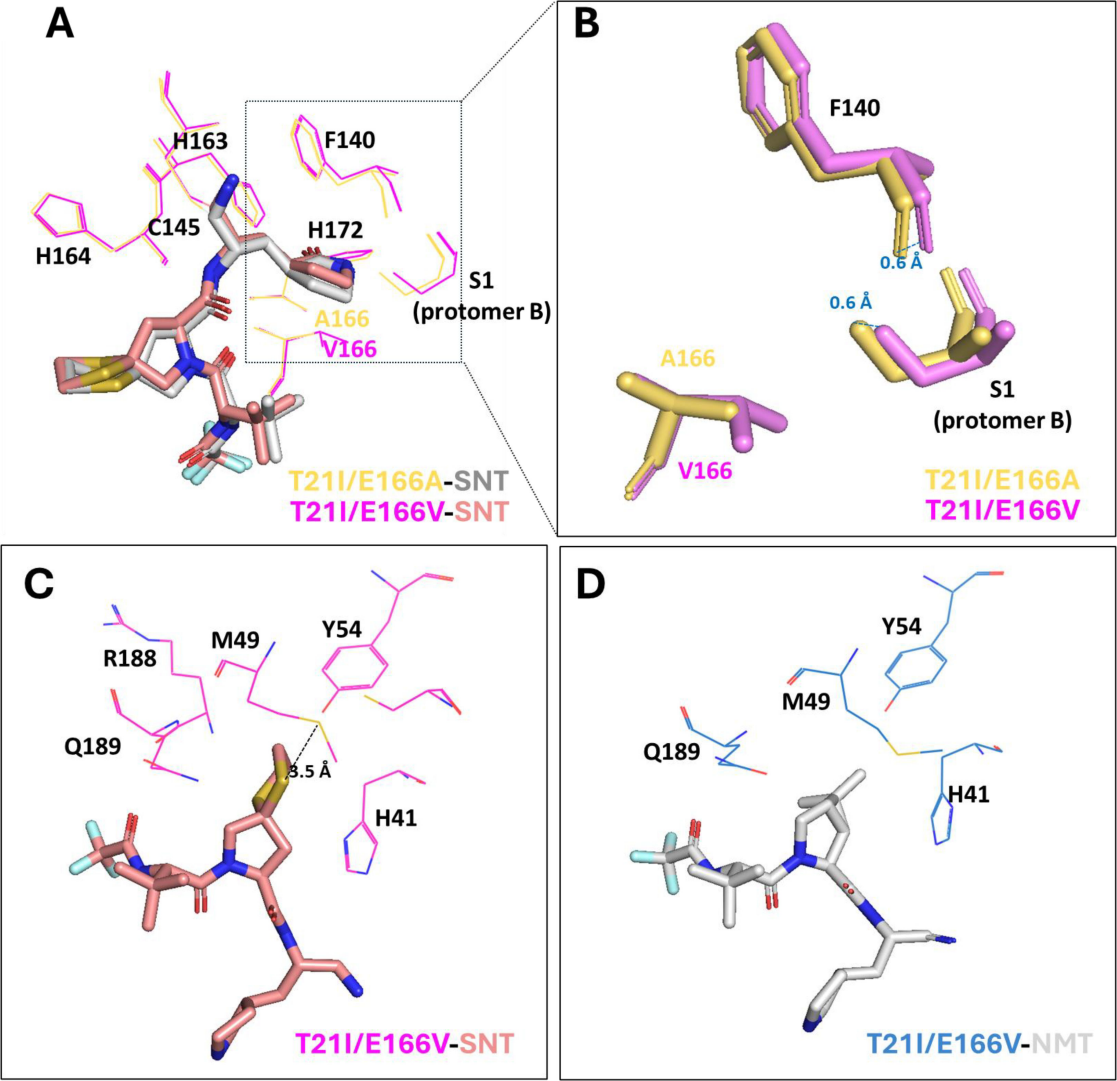
Comparison of the binding modes of SNT-T21I/E166V, SNT-T21I/E166A, and NMT-T21I/E166V (PDB: 8H51). Only catalytic pockets of 3CLpro are present in the landscapes. (**A**) Overlay of the binding modes of SNT with T21I/E166V and T21I/E166A. The residues F140, C145, H163, H164, H172, and Ser1 residue (protomer B) involved in forming hydrogen and covalent bonds are shown. (**B**) Detailed movements (blue-dashed lines) of F140 and Ser1 residue (protomer B) impacted by valine substitution compared to their positions with alanine substitution. (**C-D**) Interactions between the P2 segment of SNT (**C**) or NMT (**D**), and the S2 subsite residues of T21I/E166V. The black dashed line indicates the S-S interaction at 3.5 Å established between the two sulfur atoms.

To investigate the molecular mechanisms underlying the differential resistance of the SARS-CoV-2 3CLpro^T21I/E166A^ to these inhibitors, we determined the crystal structures of the 3CLpro^T21I/E166A^ in complex with simnotrelvir, bofutrelvir, and ensitrelvir at resolutions of 2.07 Å, 2.23 Å, and 1.83 Å, respectively (Fig. 4; Table S3). Structural comparisons of the complexes and their corresponding wild-type counterparts revealed that T21I is distal to the active site, whereas E166A resides within the S1 subsite of the active site (Fig. S3). Notably, E166A disrupts the H-bond between E166 (protomer A) and Ser1 residue (protomer B), leading to significant conformational changes in the Ser1 residue and causing its deeper intrusion into the S1 subsite of protomer A. These structural alterations in the S1 subsite significantly affect the binding mode of simnotrelvir (Fig. 4A and B). In the wild-type SARS-CoV-2 3CLpro, the P1 γ-lactam ring of simnotrelvir or bofutrelvir forms three favorable H-bonds with E166, F140, and H163 (Fig. 4A and C). Substitution of E166 with alanine eliminates the H-bond with E166, causing the P1 γ-lactam rings to form a new H-bond with the Ser1 residue of protomer B (Fig. 4B and D). This rebuilding of the H-bond network around the γ-lactam ring, disrupting H-bond interactions with the oxyanion loop and likely reducing its ability to form a covalent bond with the catalytic C145, is similar to that of nirmatrelvir-3CLpro^E166V^ reported previously. In order to investigate whether there is any difference between E166A- and E166V-simnotrelvir, we additionally gained the structure of T21I/E166V-simnotrelvir for comparison (Fig. 5 and Fig. S7). In the T21I/E166V-simnotrelvir mode, the H-bonds formed around the P1 were disrupted like the situation in the T21I/E166A-simnotrelvir complex; however, the additional -CH_3_ of valine substitution pushed F140 and Ser1 residue of protomer B away from the γ-lactam ring in approximately 0.6 Å, resulting in the strength of interactions becoming weaker than that in alanine substitution (Fig. 5A and B). On the other hand, the S-S interaction between the P2-dithiaspiro-proline and M49 was retained, resulting in more hydrophobic interactions formed among the P2 segment of simnotrelvir and the surrounding residues compared to the hydrophobic interactions in the S2 subsite of T21I/E166V-nirmatrelvir (Fig. 5C and D). For ensitrelvir, the alanine substitution results in a shift in its binding mode, with notable changes in the position of the phenyl ring occupying the S2 subsite pocket. Concomitant conformational changes in the S4 loop diminish hydrophobic interactions, collectively reducing binding affinity (Fig. 4E and F). These combined effects explain the reduced potency of ensitrelvir against the amino acid substitutes. Taken together, the crystal structures provide insight into the molecular mechanisms underlying the resistance of T21I and E166A to protease inhibitors. Importantly, structure comparison indicates that E166V induces more steric effect compared to E166A in the S1 subsite, conferring more resistance to 3CLpro.

### Different degrees of resistance result from both non-covalent and covalent reactions

**Fig 6:**
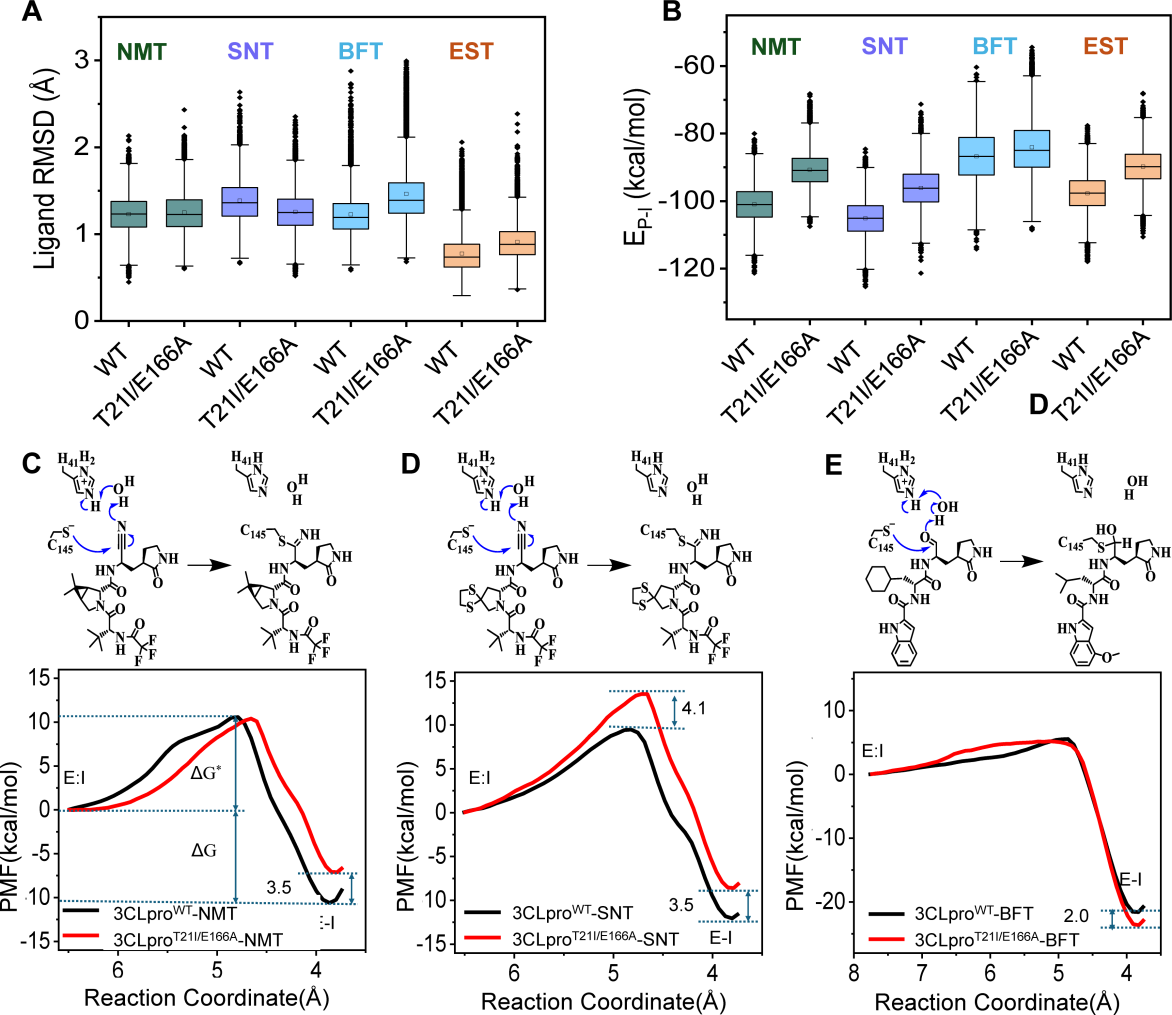
The impact of T21I/E166A mutations of 3CLpro on the non-covalent binding and covalent reactions of various inhibitors. (**A and B**) The ligand RMSDs of the four inhibitors (nirmatrelvir, simnotrelvir, bofutrelvir, and ensitrelvir) in the non-covalently bound complexes and the comparison of their non-covalent binding energies (the electrostatic and van der Waals (vdW) energies) toward wild-type 3CLpro and 3CLpro^T21I/E166A^ as measured by MD simulations. (**C–E**) The potentials of mean force (PMF) profiles for covalent reactions of nirmatrelvir, simnotrelvir, and bofutrelvir toward wild-type 3CLpro and 3CLpro^T21I/E166A^ as measured by QM/MM simulations. The schematics indicating the proposed reaction mechanisms for the three covalent inhibitors are presented.

Although shifts in molecular positioning were observed within complex structures, the binding modes alone do not fully account for the differential susceptibility of the T21I/E166A variant to simnotrelvir, bofutrelvir, and ensitrelvir. To further elucidate the differential inhibitory effects of protease inhibitors on the 3CLpro wild-type and the 3CLpro^T21I/E166A^ at the molecular level, we conducted computational simulations to examine the binding interactions of simnotrelvir, bofutrelvir, and ensitrelvir within the active sites of both enzyme variants. We first performed 500-ns molecular dynamics (MD) simulations to evaluate the 3CLpro‒inhibitor non-covalent binding interaction. In all non-covalently bound complexes (no protein-inhibitor covalent bond is formed), the time series of the root-mean-square deviation (RMSD) of the protein keeps low and steady, and the RMSDs of the inhibitors are also low, indicating the stability of the system and all inhibitors stay steady (Fig. 6A). The protease-inhibitor non-bonded interaction energy (E_P-I_) analyses exhibit that nirmatrelvir, simnotrelvir, and ensitrelvir all have weaker non-covalent binding strength (more positive E_P-I_) with the 3CLpro^T21I/E166A^ as compared to the 3CLpro wild-type, whereas the non-covalent binding of bofutrelvir is trivially influenced by the T21I and E166A (Fig. 6B).

Next, we extracted the most populated structures from MD simulations of the non-covalent complexes and submitted them for subsequent quantum mechanics/molecular mechanics (QM/MM) MD simulations to investigate the effects of T21I/E166A mutation on the covalent reactions of nirmatrelvir, simnotrelvir, and bofutrelvir (Fig. 6C through E). The combination of various atomic distances, also called collective variables (CVs), involved in the reaction was used to define the reaction coordinate. The schematics of the CVs can be seen in Fig. S4A through C. T21I and E166A destabilize the covalent reaction products of nirmatrelvir and simnotrelvir (E-I) and increase the free energy barrier for the reaction of simnotrelvir. As a result, the covalent reaction is more difficult to occur for nirmatrelvir and simnotrelvir in the 3CLpro^T21I/E166A^ as compared to the wild-type protease. In contrast, covalent inhibitor bofutrelvir, with a significantly lower free energy barrier and larger free energy difference of the reaction product (E-I) relative to the reactant (E:I), as compared to nirmatrelvir and simnotrelvir, undergoes only a trivial influence by T21I and E166A substitutes (Fig. S4D through I). Combining the MD and QM/MM MD simulation results, we could see that both non-covalent binding and covalent reaction of nirmatrelvir and simnotrelvir are impaired, whereas those of bofutrelvir are not affected by T21I and E166A. It can be thus predicted that nirmatrelvir and simnotrelvir should weaken, but bofutrelvir should remain the inhibitory potency in the 3CLpro^T21I/E166A^. For the non-covalent inhibitor ensitrelvir, it has a weaker non-covalent binding strength in the 3CLpro^T21I/E166A^ as compared to 3CLpro-WT, corresponding to a weakened inhibitory potency in the mutant. All these results are consistent with the T21I and E166A induced changes of experimental IC_50_ data (Fig. 3A through D) for all inhibitors.

### SARS2-T21I/E166A is sensitive to simnotrelvir and bofutrelvir *in vivo*

**Fig 7:**
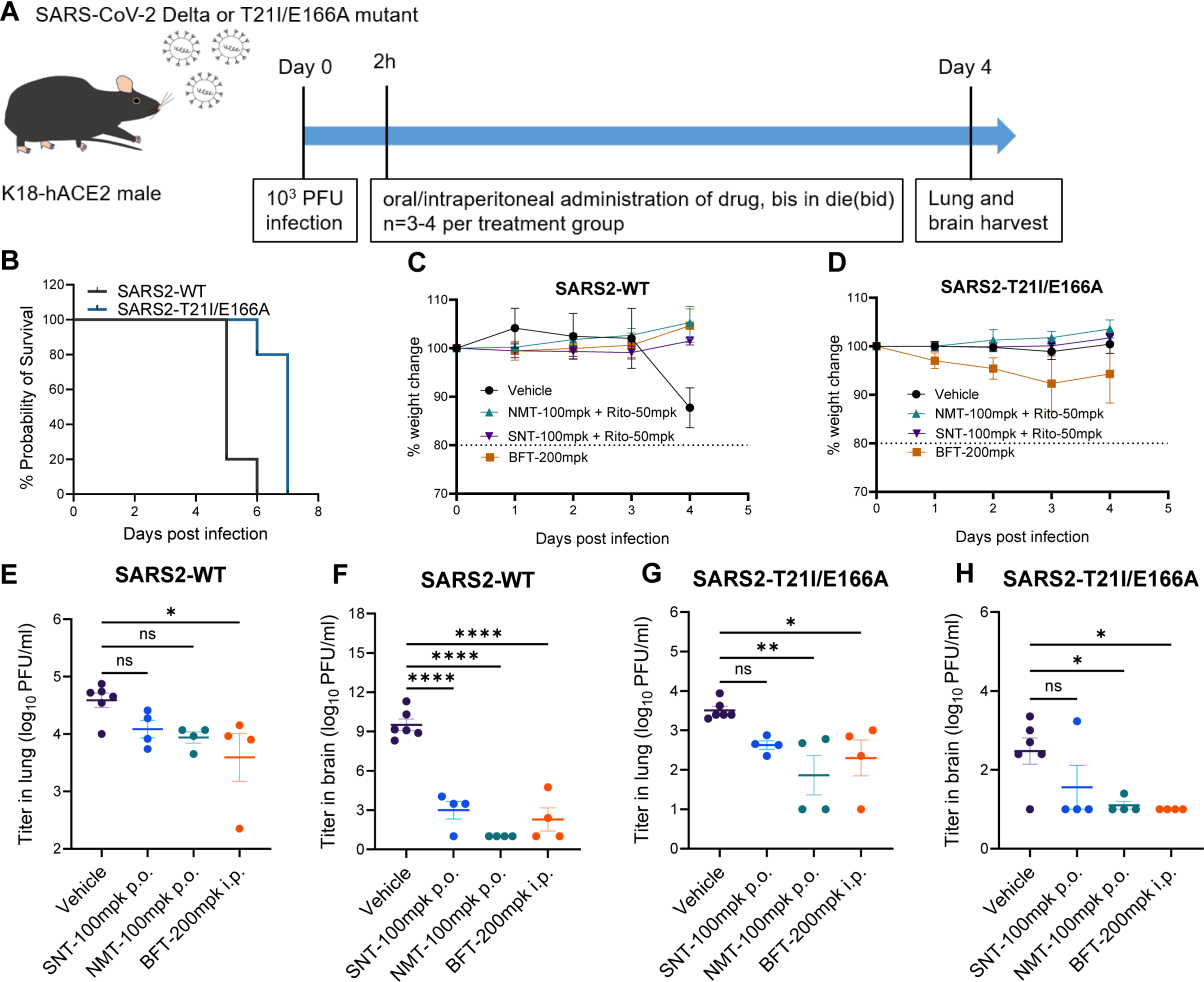
Efficacy of simnotrelvir, nirmatrelvir, and bofutrelvir against SARS2-WT and SARS2-T21I/E166A in the K18-hACE2 mouse model. (**A**) Virus propagation in the lungs and brain of mice. The mice (*n* = 4–5) were intranasally inoculated with 10^3^ PFU of the indicated viruses, and the lungs and brain were collected at 44 dpi to detect the number of viral copies. (**B**) The survival curves of mice infected with SARS2-WT or SARS2-T21I/E166A at the same dose (10^3^ PFU per mouse). (**C, D**) The body weight changes in the mice (*n* = 5) treated with vehicle, SNT, NMT, or BFT. (**E–H**) The lung and brain tissues of each group were collected on Day 4 for viral titer. The points indicate data from individual mice. SNT, simnotrelvir; NMT, nirmatrelvir; BFT, bofutrelvir. The viral titer among groups was statistically analyzed with one-way ANOVA. *P < 0.05; **P < 0.01; ***P < 0.001; ****P < 0.0001; and ns, not significant.

Subsequently, we sought to elucidate the pathogenicity of the SARS-CoV-2 T21I/E166A variant in K18-hACE2 transgenic mice, as well as to evaluate the therapeutic efficacy of simnotrelvir and bofutrelvir against infections caused by resistant viral strains. Mice were intranasally inoculated with 10^3^ PFU of SARS2-WT or SARS2-T21I/E166A and were treated with nirmatrelvir or simnotrelvir at 100 mg/kg twice daily for 4 consecutive days (Fig. 7A). The mice infected with SARS2-WT had an 80% mortality on Day 5; in contrast, the SARS2-T21I/E166A-infected mice had a 20% mortality on Day 6 (Fig. 7B). Compared with the SARS2-WT-infected mice, the SARS2-T21I/E166A-infected mice presented no significant weight loss until Day 4 (Fig. 7C and D). In mice, no matter infected with SARS2-WT or SARS-T21I/E166A, simnotrelvir (100 mg/kg plus 50 mg/kg ritonavir, p.o.), nirmatrelvir (100 mg/kg plus 50 mg/kg ritonavir, p.o.), and bofutrelvir (200 mg/kg, i.p.) administrations could effectively reduce the virus titers in the lungs and brains compared to those of the vehicle-administered mice (Fig. 7E through H). The remaining *in vivo* efficacy of the protease inhibitors to the SARS2-T21I/E166A implies that the simnotrelvir-selected resistant strain poses a limited threat in clinical therapy via simnotrelvir or nirmatrelvir in standard treatment doses.

### Bofutrelvir showed higher activity than simnotrelvir, nirmatrelvir, and ensitrelvir against T21I/E166V, L50F/E166V, and A173V

So far, there are numerous studies revealing that other mutations beyond T21I/E166A, such as T21I/E166V, L50F/E166V, and A173V, are also frequently screened by nirmatrelvir *in vitro*, and E166V significantly reduces 3CLpro susceptibility to nirmatrelvir. To investigate and compare simnotrelvir activity to these mutations, along with nirmatrelvir, ensitrelvir, and bofutrelvir, we evaluated the activity of these inhibitors against the resistance mutations while they were not present in our *in vitro* selection. The results of enzymatic assays showed that T21I/E166V and L50F/E166A conferred strong resistance to simnotrelvir, nirmatrelvir, and ensitrelvir, with IC_50_ change folds (compared to WT) of 346-fold to 369-fold, 1,142-fold to 1,302-fold, and 37-fold to 146-fold, respectively (Table S4), demonstrating that E166V has much more impact than E166A for 3CLpro susceptibility to these inhibitors. Importantly, bofutrelvir exhibited relatively much higher activity against T21I/E166V, L50F/E166V, and A173V, with the IC_50_ values in nanomoles (Table S4). The Ki values reflected similar outcomes that bofutrelvir had higher activity against the E166V-related mutations (Fig. S5).

## DISCUSSION

Although both simnotrelvir and nirmatrelvir are peptidomimetics that covalently bind at the 3CLpro active site through interactions with Cys145, the P2 of simnotrelvir is a unique dithiaspiroproline, which is believed to be the non-covalent binding portion capable of recognizing SARS-CoV-2 3CLpro with increased binding affinity (4). In China, simnotrelvir usage has been fully approved for treating COVID-19, resulting in a wide clinical coverage of simnotrelvir. Here, we evaluated and compared the resistance spectra of the wild-type SARS-CoV-2 strain in HEK293T-hACE2 cells in the presence of simnotrelvir or nirmatrelvir in a head-to-head manner. HEK293T-hACE2 cells are highly sensitive to SARS-CoV-2 infection and lack P-glycoprotein, making them suitable host cells for *in vitro* studies of peptidomimetic inhibitors. In the stepwise passage in simnotrelvir or nirmatrelvir containing medium, most of the three lineages treated with each inhibitor exhibited obvious resistance from passage 10. The results of NGS revealed that the majority of these viral lineages started from T21I and T304I mutations, which were consistent with the findings of a previous study indicating that these two mutations play a precursor role when the level of inhibitory stress is low (22). The significant resistance conferred by the E166 substitution has been widely reported in the context of passaging nirmatrelvir-adapted SARS-CoV-2 (9, 13, 22, 23). In this study, we found that SARS-CoV-2 generated similar resistance profiles in simnotrelvir and nirmatrelvir, with T21I as the precursor mutation and E166A as a late mutation. That can be explained by the similar chemical structure shared with simnotrelvir and nirmatrelvir, except the P2 segment. Interestingly, additional investigation of bofutrelvir resistance to SARS-CoV-2 revealed a distinct scenario in which only T304I accumulated in lineage 1 at passage 15, and T21I and A193T appeared at low levels in the early stages of the bofutrelvir lineages (Fig. S6). The different mutation spectra might be due to the molecular structure of bofutrelvir, which is distinct from those of nirmatrelvir and simnotrelvir. These findings suggest that the selection of SARS-CoV-2 by distinct 3CLpro inhibitors can result in different resistance developments, suggesting the feasibility of escape strategies for SARS-CoV-2 when facing various drug challenges.

To virologically study simnotrelvir and nirmatrelvir resistance to SARS-CoV-2, we obtained the resistant virus SARS2-T21I/E166A via direct plaque isolation and purification from virus populations passaged in nirmatrelvir. A cell-based assay demonstrated the significantly reduced susceptibility of SARS2-T21I/E166A to nirmatrelvir and simnotrelvir (4.3-fold to 9.2-fold); in contrast, bofutrelvir maintained activity against SARS2-T21I/E166A. Direct competition experiments revealed that SARS2-T21I/E166A had impaired growth fitness in the HEK293T-hACE2 cells, indicating that resistant strains harboring T21I/E166A have a limited ability to spread widely. However, SARS2-T21I/E166A showed a competitive advantage over SARS2-WT in culture medium containing simnotrelvir or nirmatrelvir *in vitro*, implying that drug-resistant SARS-CoV-2 potentially leads to continued infections in patients who receive long-term antiviral therapy. SARS-CoV-2 variants with E166A/V have been reported in immunocompromised patients under Paxlovid treatment (21, 24), demonstrating that the emergence of resistance mutations needs to be monitored when simnotrelvir treatment is given. Using antivirals in different categories or drug combinations might be a potential strategy to overcome SARS-CoV-2 resistance (25).

Biochemical analysis of certain 3CLpro mutants revealed that T21I/E166A mutations were responsible for the protease inhibitor resistance and growth of the authentic isolates observed in cells, with T21I/E166A conferring approximately 16-fold to 30-fold resistance to nirmatrelvir, simnotrelvir, and ensitrelvir. The resistance mechanisms of T21I and E166V in nirmatrelvir resistance have been documented in previous studies, with the H-bond network disrupted if the residue of glutamine was substituted (14). In this study, despite the properties of E166A in terms of cell-based competitive growth, nirmatrelvir resistance, and the enzyme catalytic response being similar to those of E166V previously reported, the molecular mechanisms of simnotrelvir resistance were somewhat different. The additional -CH_3_ of valine substitution pushed F140 and Ser1 of protomer B away from the γ-lactam ring in approximately 0.6 Å, resulting in the strength of interactions becoming weaker than that in alanine substitution (Fig. 5B). A previous study has proved that E166A exhibited a markedly weakened dimer with a Kd of 235 nM representing a >150-fold increase in the Kd value compared to wild-type, suggesting that E166A substitution disrupts the E166-S1 interprotomer hydrogen bond network and significantly impairs dimer stability (9). On the other hand, the S-S interaction between the P2-dithiaspiro-proline and M49 was retained, resulting in more hydrophobic interactions formed among the P2 segment of simnotrelvir and the surrounding residues compared to the hydrophobic interactions in the S2 subsite of T21I/E166V-nirmatrelvir. The difference is mainly due to the fewer -CH_3_ groups of alanine than those of valine, causing more loose space for the S2 subsite and leaving more room for the stacking of the S1 subsite and serine residue of the side chain. Although T21I is distal to the active site, T21I and L50F were previously demonstrated to be able to rescue 3CLpro activity loss of E166V through improved interactions with the substrate, resulting in resistant strains still retaining sufficient fitness (14). In K18-hACE2 mice, SARS2-T21I/E166A exhibited attenuated pathogenicity compared with the wild-type strain, with a longer mortality time and lower body weight change. Fortunately, simnotrelvir, nirmatrelvir, or bofutrelvir treatments did not show significantly reduced therapeutic effects on SARS2-T21I/E166A infections. Along with previous studies showing that the pathogenicity of resistant SARS-CoV-2 viruses harboring L50F/E166V, L50F/E166V/L167F, and M49L/E166A mutation combinations was reduced (26, 27), our results suggest that resistant viruses with E166A and T21I mutations are unlikely to spread widely.

Notably, both the resistant SARS2-T21I/E166A and T21I/E166V 3CLpro mutants presented much lower resistance to bofutrelvir. The melting temperature of 3CLpro^T21I/E166A^-bofutrelvir was significantly higher than that of 3CLpro^T21I/E166A^ bonded with nirmatrelvir, simnotrelvir, or ensitrelvir, indicating that bofutrelvir can still stabilize 3CLpro^T21I/E166A^, which may be the reason that 3CLpro^T21I/E166A^ is not very resistant to bofutrelvir. While the binding mode observed in the 3CLpro^T21I/E166A^-bofutrelvir complex structure could not explain the stability, MD simulation analysis revealed that bofutrelvir had a stronger non-covalent binding strength with 3CLpro^T21I/E166A^, which is consistent with the variance in the experimental Ki values of these inhibitors corresponding to 3CL^T21I/E166A^. Additionally, the results of the QM/MM simulation indicate that the T21I/E166A mutation destabilizes the covalent reaction products of nirmatrelvir and simnotrelvir but has a non-significant impact on the covalent reaction by bofutrelvir. Unlike simnotrelvir and bofutrelvir, ensitrelvir is a non-covalent 3CLpro inhibitor, and we found that SARS2-T21I/E166A exhibited high cross-resistance. The complex structure of 3CLpro^T21I/E166A^-ensitrelvir revealed that the crucial H-bond formed in H41 with the help of a lost water molecule, which could weaken the E-I interaction at the S2 subsite and make the H41/C145 oxyanion loop unstable.

In this study, no E166V-related mutations conferring strong resistance to 3CLpro were observed during stepwise passaging under simnotrelvir or nirmatrelvir pressures. This may be attributed to the low drug concentrations (up to 0.4 µM in the final passage) employed for achieving adapted SARS-CoV-2 strains *in vitro*. Pharmacokinetic analysis of simnotrelvir/ritonavir in humans revealed a plasma half-life (T_1/2_) of 3.1 h for simnotrelvir, with plasma concentrations declining to 1–18 μM within 36–48 h post-administration (28), whose concentration range is comparable to our *in vitro* selection concentrations. We suggest that the sustained subtherapeutic drug concentrations may persist in immunocompromised patients requiring prolonged antiviral therapy, potentially influencing resistance development. Notably, our further investigation of the protease inhibitors against other frequently reported resistant mutations revealed that simnotrelvir and bofutrelvir had better activity against T21I/E166V, L50F/E166V, and A173V compared to nirmatrelvir, which may result from the higher affinity of the P2 segment of simnotrelvir and the tighter covalent reaction contributed by the aldehyde warhead of bofutrelvir. Although previous studies have reported that E166 substitutions V/A/G/K/Q can confer 3CLpro resistance (29), the difference in resistant mechanisms among these substitutions is unclear. The binding modes of E166A- and E166V-simnotrelvir demonstrated that valine induces more steric effect compared to alanine in the S1 subsite. As a result, F140 and Ser1 residues of the other protomer that were involved in forming H-bond network around the P1 segment were pushed more away by the valine residue. This may also explain the lower activity of nirmatrelvir and bofutrelvir inhibiting T21I/E166V than inhibiting T21I/E166A.

Overall, this study found T21I/E166A mutations of SARS-CoV-2 3CLpro are selected by simnotrelvir, and the mutations confer mild to modest resistance towards simnotrelvir, nirmatrelvir, bofutrelvir, and ensitrelvir. Bofutrelvir largely maintains activity to these resistant mutations due to its aldehyde warhead. *In vivo* experiments showed that T21I/E166A retained susceptibility to simnotrelvir and bofutrelvir. In further investigation of E166V-related mutations, simnotrelvir exhibited better potency against T21I/E166V and L50F/E166V than nirmatrelvir. The resistance mechanisms were revealed via systematic methodologies, indicating different binding modes and energy barriers among these protease inhibitors and different resistance patterns between E166A and E166V. Nevertheless, there are also a few limitations in this study. First, 3CLpro is responsible for cleaving 11 sites on the viral polyprotein, but only the nsp5-nsp6 cleavage site was considered here. 3CLpro has a wide range of affinities for these cleavage sites, leading to possibly distinct resistance actions of mutations if it confronts other substrates. Second, so far, we are unclear if the resistance level of T21I/E166A causes simnotrelvir treatment failure in clinics. Moreover, it is uncertain whether other mutations, such as E166V with higher resistance levels, would emerge in simnotrelvir-adaptive passaging with longer time or another cell line. This work emphasizes that more available antiviral regimens in various categories should be developed to overcome SARS-CoV-2 3CLpro resistance.

## MATERIALS AND METHODS

### Cell and viruses

African green monkey kidney Vero E6 cells (ATCC-1586), HEK293T-hACE2-TMPRSS2 were maintained in Dulbecco’s modified Eagle’s medium (DMEM) with 10% fetal bovine serum (FBS) and 1% penicillin–streptomycin antibiotics. Cells were kept at 37°C in a 5% CO_2_ atmosphere. To select for the development of drug resistance against nirmatrelvir, simnotrelvir, or bofutrelvir, the strains Delta variant (B.1.617.2, IVCAS6.7585) were cultured in HEK293T-hACE2 cells in the presence of increasing concentrations of nirmatrelvir, simnotrelvir, or bofutrelvir and passaged 20 times. Three selections for nirmatrelvir, simnotrelvir, or bofutrelvir-resistant virus were independently performed. For each selection, Vero E6 cell and HEK293T-hACE2 cell monolayers in a 12-well plate were inoculated at a multiplicity of infection (MOI) of 0.01 with the strains Delta variant or previously passaged virus and the compound.

### Cell-based antiviral activity assay

HEK293T-hACE2 or Vero E6 cells were maintained in DMEM supplemented with 10% FBS at 37°C and humidified with 5% CO_2_. Before infection, HEK293T-hACE2 or Vero E6 cells at a density of 10^6^ cells per well in 48-well plates in DMEM (10% FBS) were incubated at 37°C and humidified with 5% CO_2_. After 12 h, the medium was replaced with 200 μL of DMEM (2% FBS) per well containing the inhibitor at a suitable concentration gradient to incubate for 2 h, then the Delta variant, SARS2-T21I/E166A, or SARS2-T304I was added at an MOI of 0.01, and then, the plates were incubated at 37°C and humidified 5% CO_2_. At 24 h post-infection, the supernatants were collected, and the viral RNA in the supernatants was extracted. For determining the viral copies, absolute quantitative RT-PCR was performed with the one-step qRT-PCR (Vazyme). All experiments involving SARS-CoV-2 were conducted in the BSL3 facility of the Wuhan Institute of Virology, Chinese Academy of Sciences. Three independent experiments of each compound determining EC_50_ values were performed, and EC_50_ values were fitted and calculated using GraphPad Prism software version 10 (GraphPad Software Inc., San 608 Diego, CA).

### Cloning, protein expression, and purification

The original expression vector (PET-28b) of 3CLpro, with a cleavable N-terminal His-tag and sumo-tag, was transformed into *Escherichia coli* BL21 (DE3) cells. The expression vectors of 3CLpro mutants were generated using site-directed mutagenesis. The bacteria were cultivated in Luria broth medium containing 0.1 mg/mL kanamycin, and protein expression was induced by adding IPTG to a final concentration of 1 mM at 16 ℃ overnight. The cells were collected by centrifugation at 8,000 × *g* for 15 min, and cell pellets were then resuspended in lysis buffer (20 mM Tris-HCl, pH 8.0, 300 mM NaCl). The cells were lysed by ultrasonication disintegration and then clarified by centrifugation at 8,000 × *g* for 30 min and filtration with a 0.45 μm filter membrane. The supernatant was loaded onto a Ni-NTA affinity column (GenScript) and washed with lysis buffer containing 20 mM imidazole. The His-tagged 3CLpro was eluted with lysis buffer containing 300 mM imidazole and displaced with the buffer (without imidazole) through a 10 KDa concentration tube. Then, the C-terminal His-tag was removed by incubation with SUMO protease at 4°C overnight. Next, the protein sample was loaded onto a Ni-NTA affinity column again, and 3CLpro with native C termini was collected in the flowthrough.

For protein crystallization, the cDNA of wild-type 3CLpro of SARS-CoV-2 (GenBank: MN908947.3) with an N-terminal SUMO tag was cloned into a pET-15b vector with *E. coli*. codon optimization. Using this plasmid as a template, the T21I/E166A mutant construct was gained by site-directed mutagenesis PCR. The plasmid was then transformed into *E. coli*. BL21 (DE3) cells for protein expression. The cell pellets were resuspended, lysed, and centrifuged. Then, the supernatants were loaded onto a Ni-NTA column (GE Healthcare), and the protein was eluted. The eluted protein samples were processed by SUMO-specific peptidase 2 (SENP2) at 4°C to remove the SUMO tag. The processed protein was further purified by Q-Sepharose followed by size-exclusion chromatography (GE Healthcare). The purified 3CLpro was stored in a solution of 10 mM Tris, pH 7.5 for protein crystallization.

### Enzymatic assays

For IC_50_ measurements, 2 μg recombinant 3CLpro wild-type or mutant proteins were incubated with a series concentration of nirmatrelvir, simnotrelvir, and bofutrelvir in 100 μL of reaction buffer at 37°C for 30 min, and the reaction was initiated by adding optimized concentrations of 3CLpro FRET substrate (MCA-AVLQSGFR-Lys (Dnp)-Lys-NH_2_) (Beyotime, Cat#P0313). The reaction was monitored for 36 min, and the initial velocity was calculated for the first 15 min by linear regression. The IC_50_ was determined by plotting the initial velocity against various concentrations of the compounds using the following equation: (Y = 100/ (1+10^((LogIC_50_-X)*HillSlope)), X = log of inhibitor concentration; Y = normalized enzyme velocity) in Prism 10 software.

For Km/Vmax measurement, proteolytic reactions were carried out with 2 μg WT or mutant proteins and series concentrations of FRET substrate (0, 0.25, 0.5, 1, 2, 3, 4, and 5 μM) in 100 μL of reaction buffer. The reaction was monitored for 1 h, and the initial velocity of the proteolytic activity was calculated by linear regression for the first 15 min of the kinetic progress curves. The initial velocity was plotted against the FRET substrate concentrations using the classic Michaelis-Menten equation (Y = Vmax*X/(km+X), X = substrate concentration; Y=enzyme velocity) in GraphPad Prism 10 software.

For inhibition constant (Ki) measurement, 2 μg WT or mutant proteins were added to various concentrations of 3CLpro FRET substrate (0, 0.25, 0.5, 1, 2, 3, 4, and 5 μM) with various concentrations of nirmatrelvir, simnotrelvir, and bofutrelvir in 100 μL of reaction buffer at 37°C to initiate the proteolytic reaction. The reaction was monitored for 1 h, and the initial velocity of the proteolytic activity was calculated by linear regression for the first 15 min of the kinetic progress curves. The Ki was calculated by plotting the initial velocity against inhibitors using the competitive inhibition equation in GraphPad Prism 10 software.

### Thermal stability assay

The binding of nirmatrelvir, simnotrelvir, or bofutrelvir to SARS-CoV-2 3CLpro mutants was monitored by differential scanning fluorimetry using a QuantStudio 6 pro Real-Time PCR System (Thermo Fisher) as previously described with minor modifications; 6 μM of WT or its mutants were preincubated with 6 μM nirmatrelvir, simnotrelvir, or bofutrelvir in reaction buffer at 16°C for 1 h. The SYPRO orange dye was added to a final concentration of 5×. The final reaction volume was 20 μL. The fluorescence of the tube was monitored under a temperature gradient range from 25°C to 94°C, with a 0.5°C/min incremental step. The melting temperature (Tm) was calculated as the mid log of the transition phase from the native to the denatured protein using a Boltzmann model in protein Thermal Shift Software v1.3. ΔTm was calculated by subtracting the reference melting temperature of proteins in the presence of DMSO from the Tm in the presence of compounds. The reported ΔTm values were averages of eight replicates.

### *In vivo* experiments in K18-hACE2 mice

Age of 7–8 weeks, K18-hACE2 male mice were purchased from Jiangsu GemPharmatech Biotechnology Co., Ltd. (Jiangsu, China). The animal experiments conformed to the use and care of laboratory animals and were approved by the ethics committee of Wuhan Institute of Virology, CAS (Approval Number: WIVAF25202201). Viral infections were performed in a biosafety level 3 (BSL-3) facility. For the survival curve analysis, five mice were intranasally infected with 50 µL 1 × 10^3^ PFU/mL of SARS-CoV-2 wild-type Delta and SARS2-T21I/E166A, respectively, then observed and recorded the physical situations of each mouse every day until dead or weight loss reached 20%. To evaluate the inhibitory effect of nirmatrelvir, simnotrelvir, and bofutrelvir on SARS2-WT and SARS2-T21I/E166A, mice were divided into four groups (*n* = 4 for each group) as follows: (i) oral vehicle treatment, (ii) oral 100 mg/kg nirmatrelvir plus 50 mg/kg ritonavir treatment, (iii) oral 100 mg/kg simnotrelvir plus 50 mg/kg ritonavir treatment, and (iv) intraperitoneal injection 200 mg/kg bofutrelvir. Mice were intranasally infected with 50 µL 1 × 10^3^ PFU/mL of SARS-CoV-2 wild-type Delta and SARS2-T21I/E166A for respective groups, and 2 h after viral infection, mice were orally treated with vehicle and drugs according to group description as described above (day 0). Mice were treated twice at 8 h intervals daily in the following days. At day 4, the mice of each group were sacrificed, and the lung and brain tissues were collected for viral copy detection and pathology analysis. Viral RNA from the lung tissues was extracted using the RNeasy Mini Kit (Qiagen) and reverse-transcribed (PrimeScript Reverse Transcriptase, Takara) according to the operation instruction; then, the absolute viral RNA copy in the tissue was detected quantitatively by real-time fluorescence quantitative PCR. The viral RNA copy was calculated by standard plasmid concentration. For histological examination, mouse lungs and brains were collected directly after euthanasia and placed in 4% paraformaldehyde for >5 days, after which tissues were embedded in 3.5-mm paraffin. Fixed tissue samples were used for hematoxylin and eosin (H&E) stain, and the image information was collected using a Pannoramic MIDI system (3DHISTECH, Budapest) and FV1200 confocal microscopy (Olympus).

### Statistical analysis

The IC_50_ and EC_50_ values were determined using nonlinear regression in GraphPad Prism software version 10 (GraphPad Software Inc., San 608 Diego, CA). The significant difference analysis of group comparisons *in vitro* and *in vivo* experiments was calculated with Student’s *t*-test and one-way analysis of variance (ANOVA), respectively.

### Crystallization, data collection, and structure determination

To obtain the complex of SARS-CoV-2 3CLpro^T21I/E166A^ mutant bound to inhibitors, 9 mg/mL of the protein was incubated with 2–4 mM inhibitors for 2 h at 0°C. Crystallization was performed at 20°C using the hanging drop vapor-diffusion method by mixing 1.2 μL protein solution plus 1.2 μL reservoir. Crystals of the complex were obtained under the condition of 4%–20% PEG6000, 100 mM MES (pH 5.5-7.0), and 3% DMSO. The crystals were transferred to a cryoprotectant solution (reservoir solution supplemented with 20% glycerol) and flash-frozen in liquid nitrogen. The diffraction data were collected at 100 K with a wavelength of 0.97,923 Å on beamline BL19U1 (30) at the Shanghai Synchrotron Radiation Facility. The data were processed using XDS (31) and scaled using Aimless (32). The complex structures were determined by molecular replacement using the program CCP4 (33) with a search model of PDB code 6M2N (34). The model was rebuilt using Coot (35) and refined with the program PHENIX (36). The final complex structures were manually refined with Coot and PHENIX. The diffraction data set and statistics of final refinement are summarized in Table S3.

### Molecular dynamics simulation

The wild-type (WT) or T21I/E166A mutated SARS-CoV-2 3CLpro non-covalently complexed with various inhibitors (nirmatrelvir, simnotrelvir, bofutrelvir, and ensitrelvir) were simulated by using classical molecular dynamics (MD) simulations. The atomic coordinates of the WT 3CLpro non-covalent complex systems were achieved from the crystal structures reported previously: WT 3CLpro–nirmatrelvir (7RFW) (37), WT 3CLpro–simnotrelvir (8IGX) (4), WT 3CLpro–bofutrelvir (6LZE) (38), and WT 3CLpro–ensitrelvir (8HBK) (14), with the inhibitors and crystal waters maintained, whereas other solvent molecules were removed. The atomic coordinates of 3CLpro^T21I/E166A^-inhibitor complexes were obtained from the crystal structures as measured by accompanying X-ray crystallography experiments, except that the 3CLpro^T21I/E166A^-nirmatrelvir (without available crystal structure) was prepared by manually mutating the 166 residue in the reported 3CLpro^T21I/E166V^-nirmatrelvir crystal structure (8H51) (14). For all covalent inhibitors, the formed 3CLpro-inhibitor covalent bond in each crystal structure was removed, and the unreacted warhead was recovered manually using GaussView.

The protonation states of all titratable residues except the catalytic dyad of H41 and C145 in SARS-CoV-2 3CLpro were evaluated at pH 7.5 using Schrodinger suite software. All residues were found in their standard protonation states. After a detailed inspection of the environment surrounding each histidine residue, all evaluated histidines were neutral, among which H64 and H80 were protonated in the Nδ position, while the remaining H163, H164, H172, and H246 were protonated on Nε. The H41 and C145 were in an ion pair state.

Each simulated system was solvated in a cubic box filled with a total of ~20,000 water molecules, in which multiple Na^+^/Cl^-^ ions were added to neutralize the protein charges. AMBER 22 suite of programs (39) was employed for simulations with the FF14SB force field (40) for proteins and TIP3P model for water molecules. The inhibitors were modeled using generalized AMBER force field (GAFF) (41) with restrained electrostatic potential (RESP) partial charges (42) fitted with Gaussian 09.

Each system was initially minimized for 50,000 steps and heated to 300 K, with the protein (and inhibitor) heavy atoms being fixed using a harmonic restraint with a force constant of 10.0 kcal mol^−1^ Å^−2^. Subsequently, the protein was relaxed by two steps of equilibrium at a constant temperature of 300 K and a constant pressure of 1 atm (*NPT* ensemble): 2 ns for the relaxing protein sidechain and 2 ns for the protein main chain. The SHAKE algorithm was used to fix all covalent bonds involving hydrogen atoms, and periodic boundary conditions were used to avoid edge effects. The Particle Mesh Ewald method was applied to treat long-range electrostatic interactions, and the cutoff distance for long-range terms (electrostatic and van der Waals (vdW) energies) was set as 10.0 Å (43). The Langevin dynamics with a collision frequency of 3.0 ps^−1^ was adopted to control the temperature. Finally, a production run was performed on each equilibrated system without constraints on any system atoms, using the GPU version of AMBER 22, and each trajectory lasted 500 ns. The atomic coordinates were saved every 10,000 steps for data analysis.

### Quantum mechanics/molecular mechanics (QM/MM) MD simulations

The MD measured stable complex structures were then clustered to be used as initial states for consequent QM/MM MD simulations to explore the free energy profiles associated with the 3CLpro-inhibitor covalent chemical reactions. In the QM region, the side chains of the catalytic dyad (H41 and C145) and a fragment of the inhibitors were involved, along with D187 and a conserved crystal water (W_cat_) in between H41 and D187. The remaining part of the system was described at the MM level. Specifically, the QM-treated fragment of each inhibitor included all atoms at P1′ and P1, and the peptidomimetic bond up to the carbonyl atom of P2 segment. In the covalent reactions for nirmatrelvir, simnotrelvir, and bofutrelvir, a water molecule in between H41 and the inhibitor warhead was also included in the QM region. As the QM region crossed covalent bonds, the QM/MM boundary was chosen to cut C-C non-polar bonds, and link atoms (hydrogens) were added automatically for the QM calculation without user intervention (44). A semi-empirical density functional theory-based tight-binding method (DFTB) potential was used to describe the QM subsystem (45), while the MM region was described by the force fields of FF14SB for protein, TIP3P for water, and GAFF for the remaining part of the inhibitors. A cutoff radius of 10 Å was used for QM/MM interactions, and the temperature was controlled at 300 K.

First, steered molecular dynamics (SMD) (46) was performed to yield the reaction path in the QM region following a specific reaction coordinate. The QM/MM SMD was run by using a harmonic force constant of 500 kcal/mol/Å^2^ to pull the system along the reaction coordinate. Then, multiple structures were evenly selected from the SMD trajectory with an increment in the reaction coordinate of ~0.15 Å as starting points for the subsequent umbrella sampling (US) simulation. A constraint was added along the reaction coordinate with an umbrella force constant of 100 kcal/mol/Å^2^ in each US window, making sure the sampled reaction path is overlapped among individual windows. In every window, a simulation was performed for 250 ps at 300 K with a time step of 1 fs. Finally, the detailed free energy profile, in terms of potential of mean force (PMF), was calculated using the Weighted Histogram Analysis Method (47).

## Data Availability

The data for this study are available by contacting the corresponding author upon reasonable request.

## SUPPLEMENTAL MATERIAL

Fig. S1 to S7; Tables S1 to S4 jvi.02223-25-s0001.docx SARS-CoV-2 3CLpro mutations T21I and E166A confer differential resistance to simnotrelvir, bofutrelvir, and ensitrelvir.

ASM does not own the copyrights to Supplemental Material that may be linked to, or accessed through, an article. The authors have granted ASM a non-exclusive, world-wide license to publish the Supplemental Material files. Please contact the corresponding author directly for reuse.
