# Supplementary material for: SARS-CoV-2 3CLpro mutations T21I and E166A confer differential resistance to simnotrelvir, bofutrelvir, and ensitrelvir
Source: J Virol. 2026 Apr 27;100(5):e02223-25. doi: 10.1128/jvi.02223-25 (PMC13185579; doi:10.1128/jvi.02223-25)
Supplement: Fig. S1 to S7; Tables S1 to S4 — SARS-CoV-2 3CLpro mutations T21I and E166A confer differential resistance to simnotrelvir, bofutrelvir, and ensitrelvir. [file jvi.02223-25-s0001.docx]

**SARS-CoV-2 3CLpro mutations T21I and E166A confer differential resistance to simnotrelvir, bofutrelvir, and ensitrelvir**

**Supplemental Files**

**Fig S1.** Responses of Omicron 3CLpro and Omicron 3CLpro^T21I/E166A^ to simnotrelvir, nirmatrelvir, bofutrelvir, and ensitrlvir via a FRET-based enzymatic assay. The data represent the means ± SDs of three independent measurements.


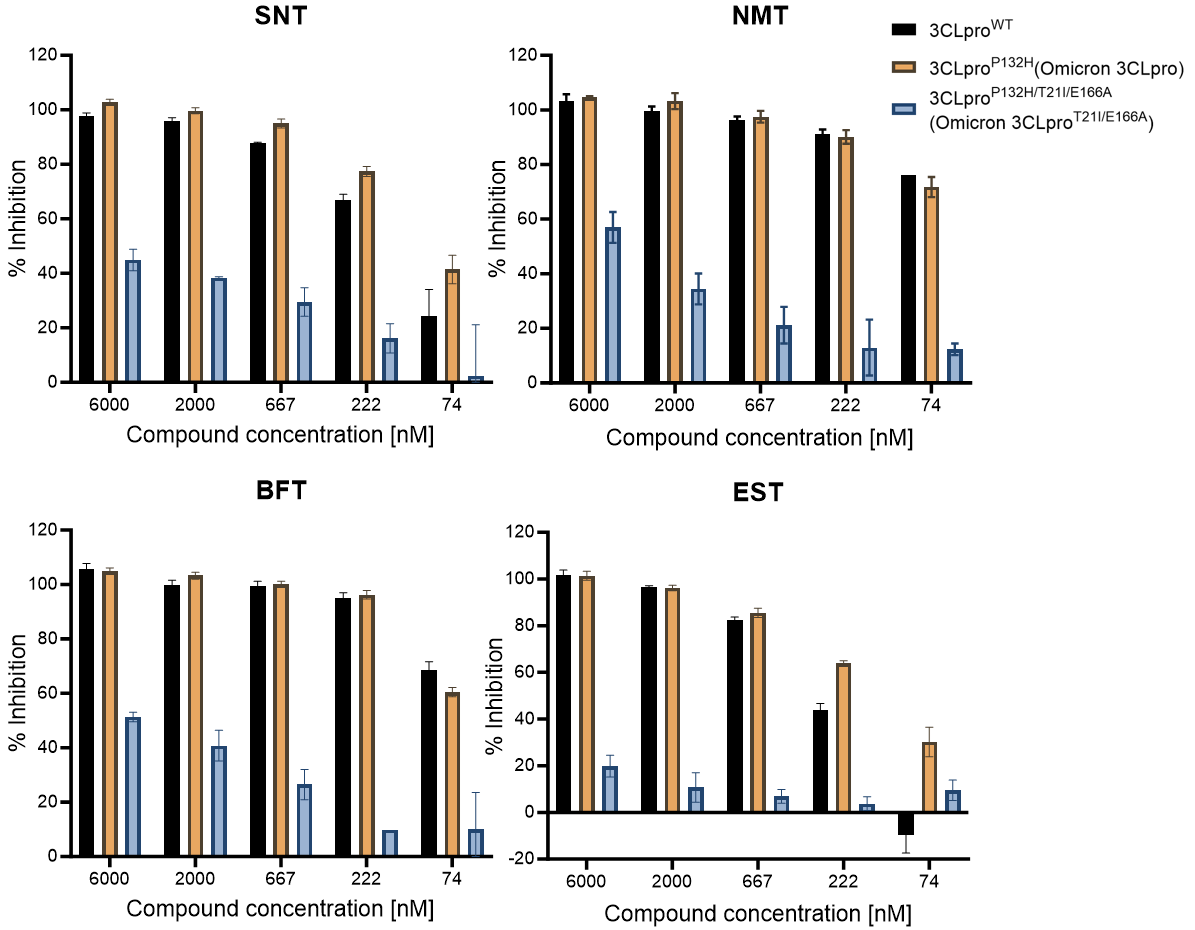


**Fig S2.** The evaluation of 3CLpro-inhibitor binding affinity via a fluorescence-based thermal shift assay.


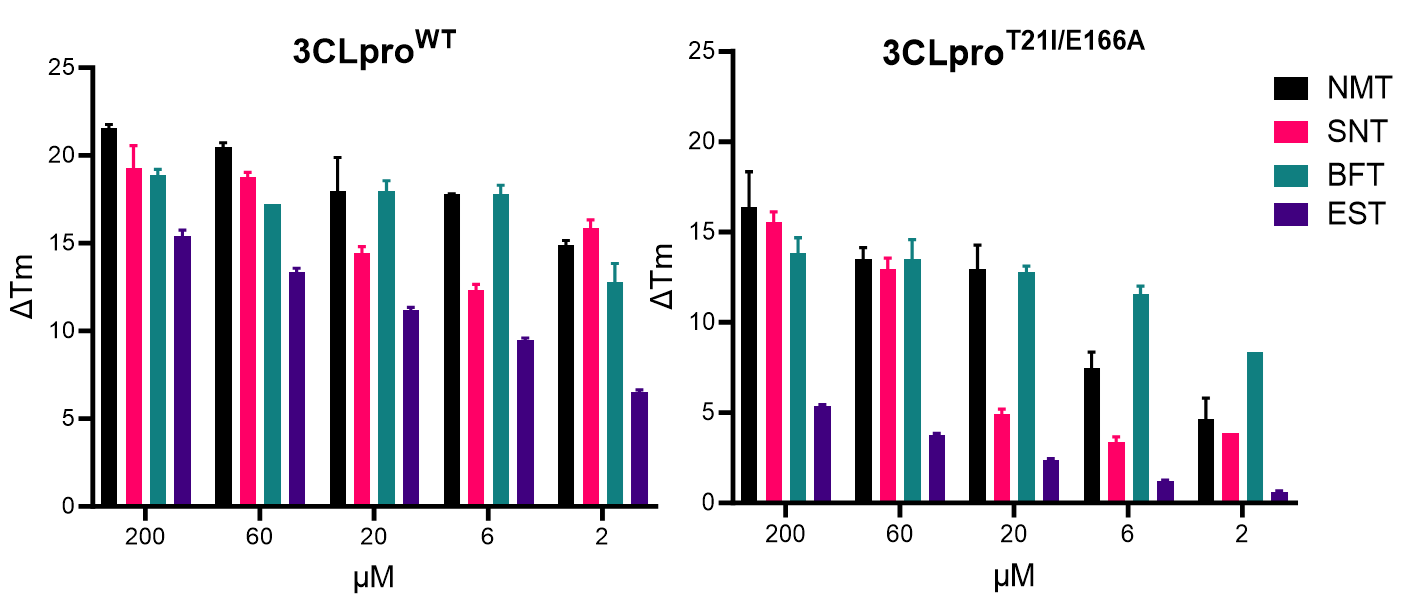


The bar indicates the standard deviation (SD) from 3–6 independent experiments.

Fig. S3: Structural comparison of SARS-CoV-2 3CLpro^T21I/E166A^ mutant–ligand complexes with their wild-type counterparts. (A) Surface representation of the 3CLpro^T21I/E166A^ mutant highlighting the S1–S4 subsites. (B–D) Overlay of SARS-CoV-2 3CLpro^T21I/E166A^ mutant in complex with simnotrelvir (B) bofutrelvir (C) and ensitrelvir (D) and their respective wild-type counterparts. Bound small molecules (simnotrelvir, bofutrelvir, and ensitrelvir) are depicted as dark green-colored sticks in the T21I/E166A mutant and slate sticks in the wild-type. Hydrogen bonds are shown as yellow dashed lines. SNT, simnotrelvir; BFT, bofutrelvir; EST, ensitrelvir.

**
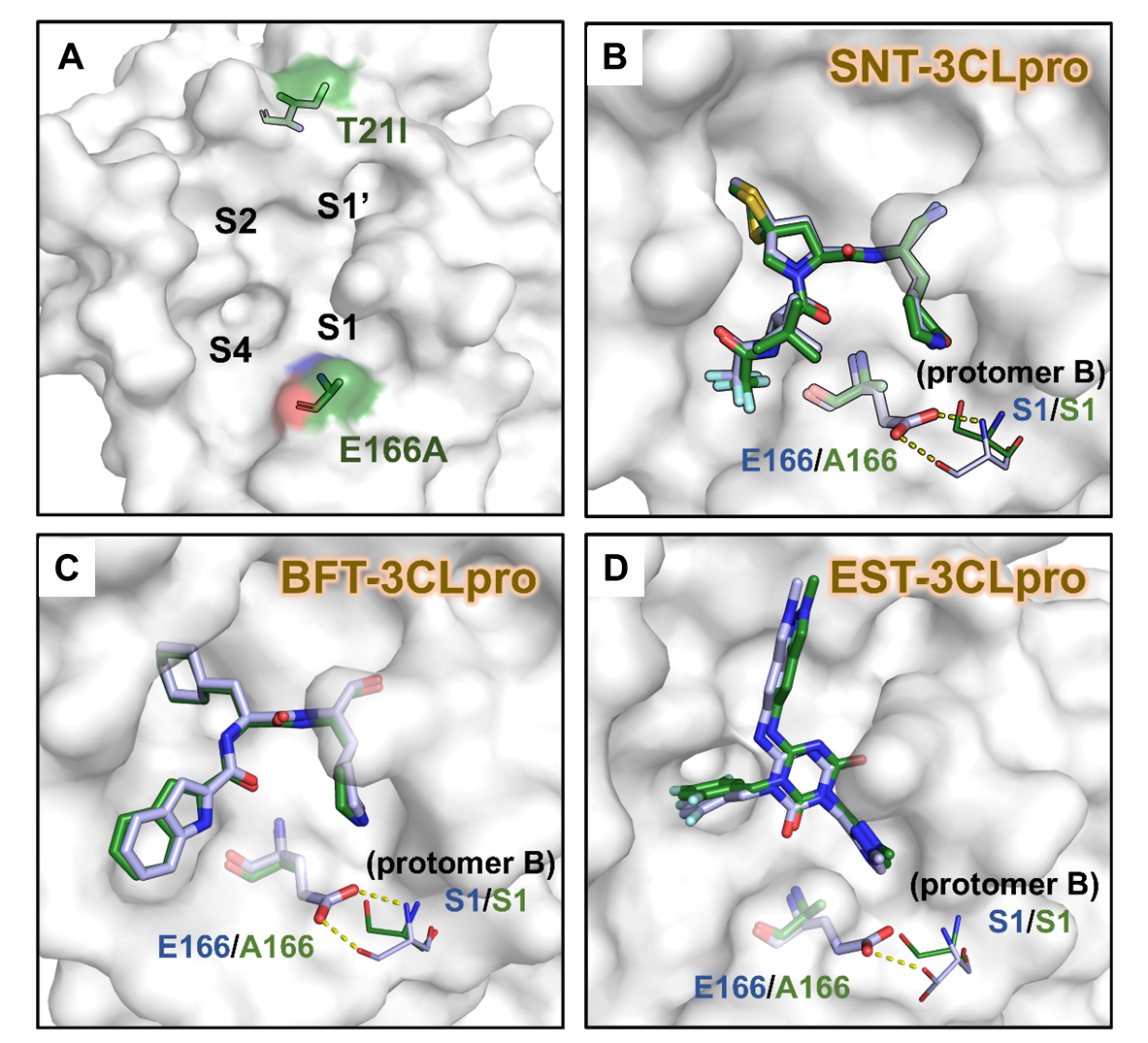
**

**Fig. S4: Evolution of the distances employed as the collective variables (CVs) along the reaction coordinates (RCs) of the covalent reactions.** (A-C) Schematic of the CVs for the reactions of nirmatrelvir, simnotrelvir, and bofutrelvir with SARS-CoV-2 3CLpro, respectively. CV1: dC145-Sγ‒P1′-C (the distance between C145-Sγ and P1′-C atoms); CV2: dP1′-N‒Water-H (the distance between P1′-N and water hydrogen) for nirnatrelvir and simnotrelvir or dP1′-O‒Water-H (the distance between P1′-O and water hydrogen) for bofutrelvir; CV3: dH41-Hε‒Water-O (the distance between H41-Hε and water oxygen). The reaction coordinate for the covalent reaction in each system is defined as: RC = CV1 + CV2 + CV3. (D-F) Evolution of the CVs along RCs for the reactions of nirmatrelvir, simnotrelvir, and bofutrelvir with wild-type 3CLpro. (G-I) Evolution of the CVs along RCs for the reactions of nirmatrelvir, simnotrelvir, and bofutrelvir with 3CLproT21I/E166A mutant. Dash line represents the position of the transition state (TS).


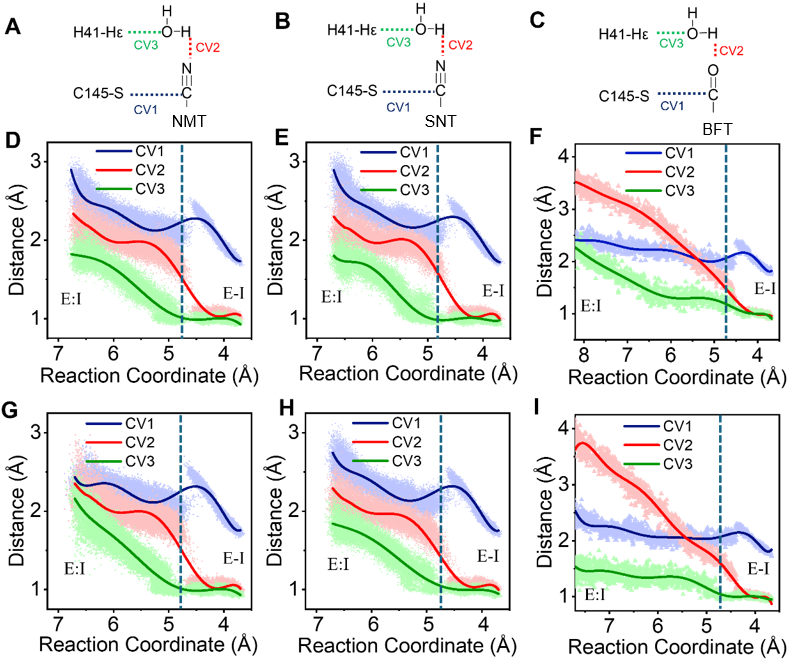


**Fig S5.** The inhibition constant (Ki) values of 3CLpro^T21I/E166V^,3CLpro^L50F/E166V^, and 3CLpro^A173V^ to simnotrelvir (SNT), nirmatrelvir (NMT), bofutrelvir (BFT), and ensitrelvir (EST) calculated from their velocities via the Morison equation. The data represent the means ± SDs of three independent measurements.


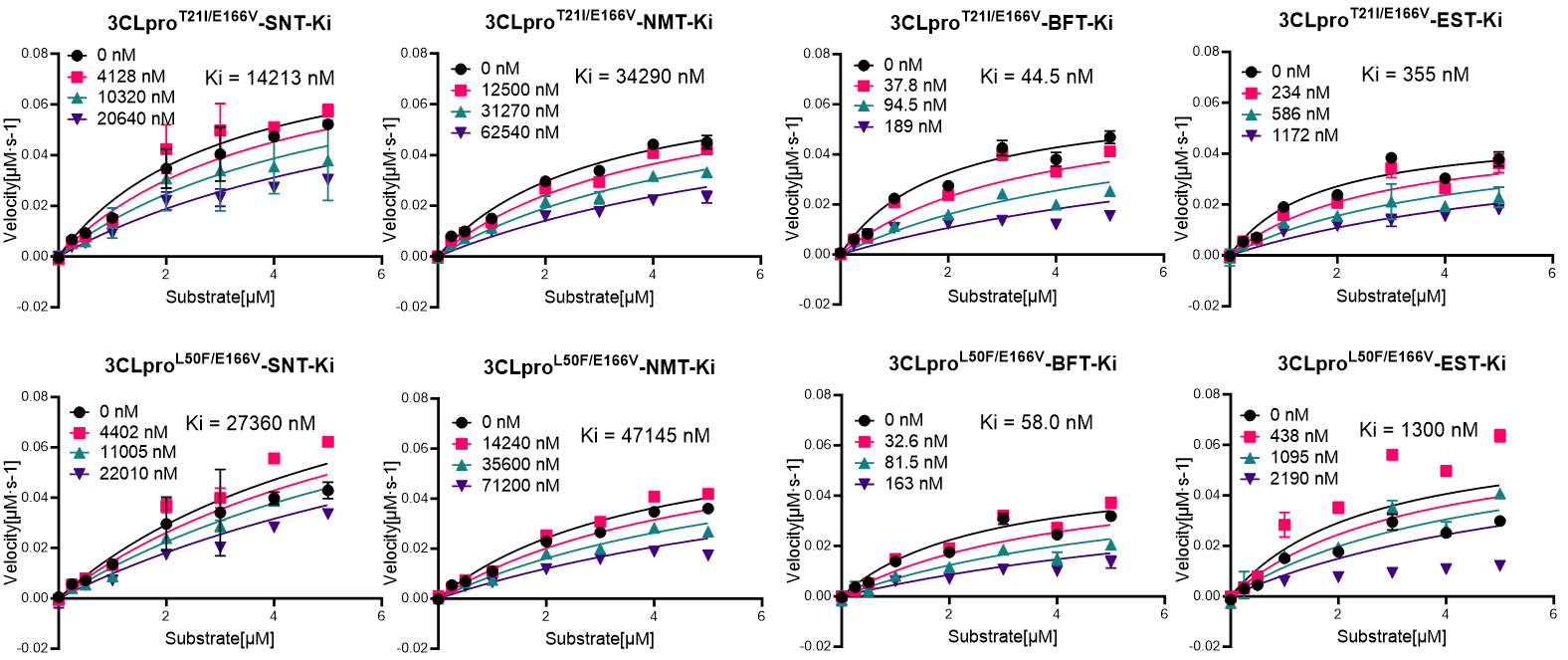


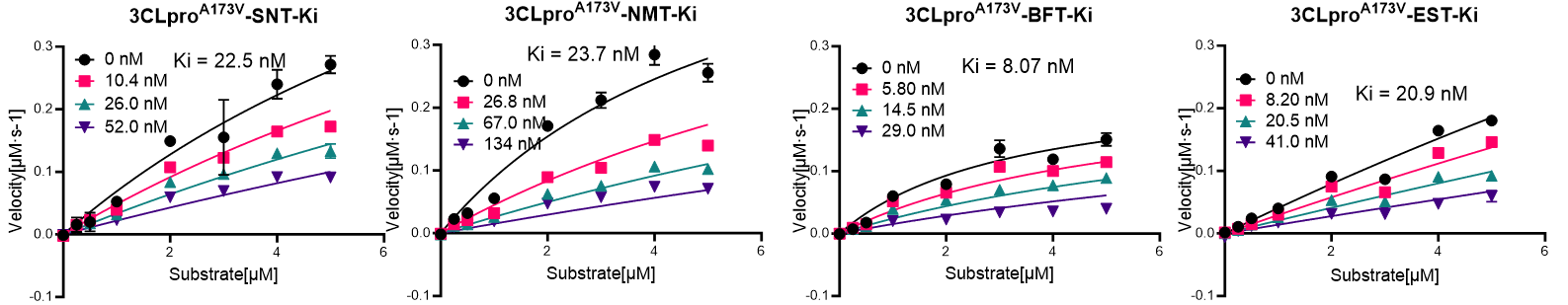


**Fig S6.** Identification of bofutrelvir resistance of SARS-CoV-2 Delta strain in HEK293T-hACE2 cells.


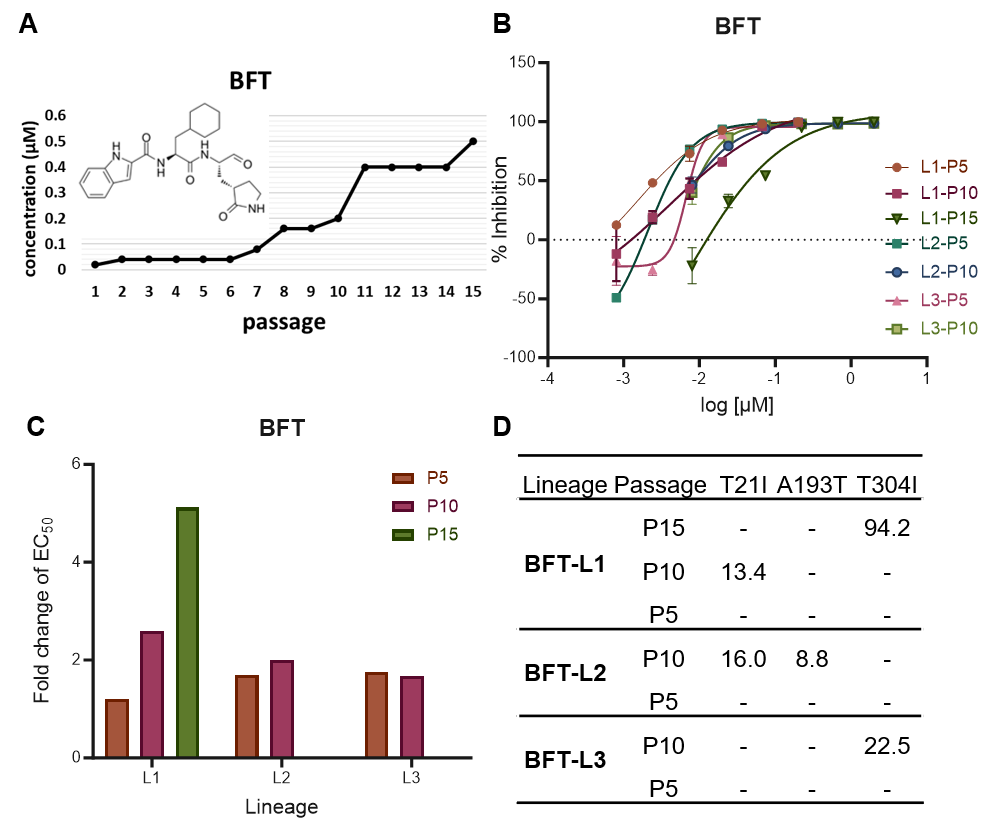


(A) The scheme of the cell-based passaging experiments for SARS-CoV-2 delta variant mutant selection in HEK293T-hACE2 cells. (B) Changes in drug response during series passages of SARS-CoV-2 with bofutrelvir. HEK293T-hACE2 cells were infected in triplicate (L1-L3) with SARS-CoV-2 and passaged to fresh cells every 3 days for 10 or 15 passages. Validation of bofutrelvir resistance for the indicated passages from each of the three lineages. (C) The fold changes of EC_50_ values of viral passages from each lineage by bofutrelvir compared to the EC_50_ values of Delta wild-type. The bars represent one of three independent experiments. (D) The amino acid mutations in 3CLpro found in the indicated passages from each lineage. The number indicates mutation frequency.

**Fig S7. 2*Fo-Fc* density maps contoured at 2.0 σ are shown for inhibitors and covalent binding residue Cys145.** SARS-CoV-2 3CLpro T21I/E166A mutant (A-C) and T21I/E166V mutant (D) are shown as green and blue cartoon, respectively. Inhibitors and residue Cys145 are shown as sticks in different colors. SNT, simnotrelvir (wheat); BFT, bofutrelvir (pink); EST, ensitrelvir (salmon).


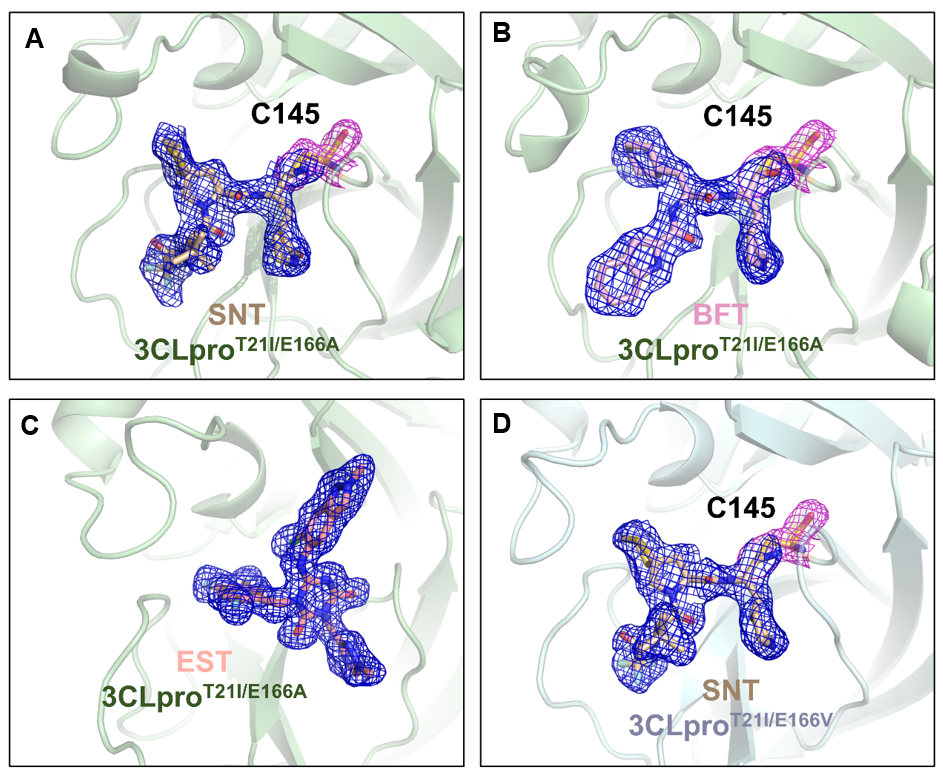


**Table S1.** Mutations were found in the whole genomes of SARS-CoV-2 passages 5-15 in nirmatrelvir and simnotrelvir by next-generation sequencing (NGS).

| Name | Genome position | REF | ALT | Gene area | Mutation |
| --- | --- | --- | --- | --- | --- |
| SNT-P5L1 | 6758 | T | G | nsp3 | C1347G |
|  | 18142 | A | G | nsp14 | T35A |
| SNT-P5L2 | 2088 | T | C | nsp2 | L428S |
|  | 23997 | C | T | S | P812L |
|  | 26281 | A | C | E | I13L |
| SNT-P5L3 | 18142 | A | G | nsp14 | T35A |
| SNT-P10L1 | 10116 | C | T | nsp5 | T21I |
|  | 10965 | C | T | nsp5 | T304I |
|  | 11601 | T | C | nsp6 | I210T |
|  | 18142 | A | G | nsp14 | T35A |
|  | 20954 | A | G | nsp16 | D99G |
|  | 22289 | G | T | S | A243S |
|  | 23997 | C | T | S | P812L |
|  | 29625 | C | T | 3’UTR |  |
| SNT-P10L2 | 2088 | T | C | nsp2 | L428S |
|  | 10116 | C | T | nsp5 | T21I |
|  | 23997 | C | T | S | P812L |
|  | 24449 | G | A | S | V963I |
|  | 26270 | C | T | E | T9I |
|  | 26281 | A | C | E | I13L |
|  | 27921 | A | G | M-N | I10V |
| SNT-P10L3 | 10116 | C | T | nsp5 | T21I |
|  | 19955 | C | T | nsp15 | T112I |
|  | 23624 | G | C | S | A688P |
|  | 23626 | T | G |  |  |
|  | 23997 | C | T | S | P812L |
|  | 25553 | C | T | S-E | A54V |
|  | 26281 | A | C | E | I13L |
|  | 26395 | C | T | E | L51F |
| SNT-P15L1 | 10116 | C | T | nsp5 | T21I |
|  | 10560 | C | T | nsp5 | T169I |
|  | 10965 | C | T | nsp5 | T304I |
|  | 11124 | C | T | nsp6 | A51V |
|  | 18182 | A | G | nsp14 | D48G |
|  | 18184 | A | G | nsp14 | M49V |
|  | 24469 | T | A | S | N969K |
|  | 25553 | C | T | S-E | A54V |
|  | 26395 | C | T | E | L51F |
| SNT-P15L2 | 311 | C | T | nsp1 | L16F |
|  | 2088 | T | C | nsp2 | L428S |
|  | 2790 | C | T | nsp3 | T24I |
|  | 7299 | C | T | nsp3 | A1527V |
|  | 8156 | T | C | nsp3 | S1813P |
|  | 10116 | C | T | nsp5 | T21I |
|  | 10551 | A | C | nsp5 | E166A |
|  | 20522 | A | G | nsp15 | D301G |
|  | 24945 | T | C | S | V1128A |
|  | 26270 | C | T | E | T9I |
|  | 26281 | A | C | E | I13L |
|  | 26709 | G | A | M | A63T |
| SNT-P15L3 | 3728 | G | T | nsp3 | G337C |
|  | 6449 | C | T | nsp3 | L1244F |
|  | 10116 | C | T | nsp5 | T21I |
|  | 10551 | A | C | nsp5 | E166A |
|  | 18142 | A | G | nsp14 | T35A |
|  | 26557 | A | G | M | E12G |
|  | 27393 | C | T | M-N |  |
|  | 27625 | C | T | M-N | R78C |
|  | 29625 | C | T | 3’UTR |  |
| NMT-P5L1 | 10116 | C | T | nsp5 | T21I |
|  | 18142 | A | G | nsp14 | T35A |
| NMT-P5L2 | 18142 | A | G | nsp14 | T35A |
| NMT-P5L3 | 18142 | A | G | nsp14 | T35A |
|  | 23997 | C | T | S | P812L |
| NMT-P10L1 | 2767 | A | C | nsp3 | Q16H |
|  | 10116 | C | T | nsp5 | T21I |
|  | 10965 | C | T | nsp5 | T304I |
|  | 16208 | C | T | nsp12 | A923V |
|  | 18142 | A | G | nsp14 | T35A |
|  | 23997 | C | T | S | P812L |
|  | 25549 | C | T | S-E | L53F |
| NMT-P10L2 | 9360 | C | T | nsp4 | T2214I |
|  | 10116 | C | T | nsp5 | T21I |
|  | 10965 | C | T | nsp5 | T304I |
|  | 12741 | C | T | nsp9 | T19I |
|  | 18142 | A | G | nsp14 | T35A |
|  | 23624 | G | C | S | A688P |
|  | 23626 | T | G |  |  |
| NMT-P10L3 | 44 | C | T | 5‘UTR |  |
|  | 936 | C | T | nsp2 | T44I |
|  | 6105 | A | G | nsp3 | Y1129C |
|  | 10116 | C | T | nsp5 | T21I |
|  | 10551 | A | C | nsp5 | E166A |
|  | 11750 | C | T | nsp6 | L260F |
|  | 18142 | A | G | nsp14 | T35A |
|  | 23997 | C | T | S | P812L |
|  | 25411 | A | C | S-E | I7L |
|  | 26281 | A | C | E | I13L |
|  | 29667 | T | C | 3’UTR |  |
| NMT-P15L1 | 256 | G | T | 5‘UTR |  |
|  | 2767 | A | C | nsp3 | Q16H |
|  | 3416 | A | G | nsp3 | K233E |
|  | 4141 | A | C | nsp3 | Q474H |
|  | 10116 | C | T | nsp5 | T21I |
|  | 10484 | T | G | nsp5 | S144A |
|  | 10551 | A | C | nsp5 | E166A |
|  | 11750 | C | T | nsp6 | L260F |
|  | 16208 | C | T | nsp12 | A923V |
|  | 19366 | C | T | nsp14 | P443S |
|  | 26558 | G | T | M | E12D |
| NMT-P15L2 | 632 | C | T | nsp1 | L123F |
|  | 3003 | A | C | nsp3 | E95A |
|  | 10116 | C | T | nsp5 | T21I |
|  | 10551 | A | C | nsp5 | E166A |
|  | 10965 | C | T | nsp5 | T304I |
|  | 12741 | C | T | nsp9 | T19I |
|  | 18142 | A | G | nsp14 | T35A |
| NMT-P15L3 | 936 | C | T | nsp2 | T44I |
|  | 10116 | C | T | nsp5 | T21I |
|  | 10551 | A | C | nsp5 | E166A |
|  | 11750 | C | T | nsp6 | L260F |
|  | 17125 | C | T | nsp13 | L297F |
|  | 18142 | A | G | nsp14 | T35A |
|  | 25411 | A | C | S-E | I7L |
|  | 26281 | A | C | E | I13L |
|  | 29667 | T | C | 3’UTR |  |
|  | 2767 | A | C | nsp3 | Q16H |
|  | 10116 | C | T | nsp5 | T21I |
|  | 10551 | A | T | nsp5 | E166A |
|  | 11750 | C | T | nsp6 | L260F |

Each passage contains three lineages (L1-L3). The genome of the SARS-CoV-2 wild type was used as a reference sequence.

**Table S2.** Mutations occurred in the whole genomes of SARS2-T21I/E166A analyzed with next-generation sequencing (NGS).

| SARS2-T21I/E166A | | |
| --- | --- | --- |
| Genome position | Gene region | Mutation |
| 3728 | nsp3 | G337C |
| 6449 | nsp3 | L1244F |
| 10116 | nsp5 | T21I |
| 10551 | nsp5 | E166A |
| 18142 | nsp14 | T35A |
| 29625 | 3’-UTR | - |

The whole genome of SARS-CoV-2 Delta wild-type strain was used as reference sequence.

**Table S3.** Crystallography data collection and refinement statistics of SARS-CoV-2 3CLpro^T21I/E166A^ and 3CLpro^T21I/E166V^ mutants in complex with inhibitors.

| PDB ID | 9KOA | 9KOB | 9KOC | 24UQ |
| --- | --- | --- | --- | --- |
| Ligand | simnotrelvir | bofutrelvir | ensitrelvir | simnotrelvir |
| 3CLpro mutants | T21I/E166A | T21I/E166A | T21I/E166A | T21I/E166V |
| Space Group | P 1 21 1 | P 1 21 1 | P 21 21 2 | C 1 2 1 |
| Cell Dimension: a (Å) | 45.75 | 46.04 | 46.18 | 113.98 |
| b (Å) | 53.31 | 53.54 | 63.41 | 53.36 |
| c (Å) | 113.27 | 112.94 | 107.01 | 46.06 |
| Wavelength (Å) | 0.97923 | 0.97923 | 0.97923 | 0.97918 |
| Reflections (unique) | 32825 | 25822 | 27852 | 16903 |
| Resolution Range (Å) | 55.62-2.07 | 55.31-2.23 | 54.55-1.83 | 30.50-2.05 |
| Highest-Resolution Shell (Å) | 2.08-2.07 | 2.36-2.23 | 1.93-1.83 | 2.12-2.05 |
| Redundancy | 6.4 (6.3) | 6.3 (6.7) | 12.7 (13.7) | 6.8 (7.0) |
| I/σ (I) | 5.5 (2.5) | 7.4 (2.9) | 9.6 (2.3) | 13.95 (1.92) |
| Highest-Resolution Shell CC_1/2_ | 0.911 | 0.870 | 0.628 | 0.717 |
| Completeness (%) | 99.7 (99.3) | 97.8 (99.7) | 98.2 (97.2) | 98.4 (97.1) |
| Rwork/Rfree | 0.2539/0.2825 | 0.2191/0.2569 | 0.2129/0.2398 | 0.2285/0.2661 |
| RMS Values | | | |  |
| Bond length (Å) | 0.005 | 0.006 | 0.010 | 0.002 |
| Bond angle (°) | 0.868 | 0.920 | 1.251 | 0.526 |
| Numbers of Non-hydrogen Atoms | | | |  |
| Protein | 4534 | 4530 | 2355 | 2273 |
| Inhibitor | 72 | 66 | 37 | 36 |
| Water Oxygen | 85 | 124 | 151 | 41 |
| Others | 0 | 0 | 0 | 0 |
| Clashscore | 1.68 | 2.24 | 1.70 | 3.56 |
| MolProbity Score | 0.92 | 1.00 | 1.16 | 1.22 |
| B-factor (Å^2^) | | | |  |
| Protein | 35.41 | 32.30 | 21.61 | 46.05 |
| Inhibitor | 43.02 | 26.82 | 17.07 | 41.94 |
| Water Oxygen | 33.80 | 32.53 | 26.65 | 40.04 |
| Ramachandran plot | | | |  |
| Favored (%) | 99.00 | 98.00 | 96.38 | 97.66 |
| Allowed (%) | 0.67 | 1.67 | 3.62 | 2.01 |
| Outliers (%) | 0.33 | 0.33 | 0.00 | 0.33 |

**Table S4.** Evaluation of the susceptibility of 3CLpro^T21I/E166V^, 3CLproL^50F/E166V^, and 3CLpro^A173V^ to four protease inhibitors in FRET-based enzymatic assay.

| Inhibitor | Protease | IC_50_ (µM) | Fold to WT | Ki (nM) | Fold to WT |
| --- | --- | --- | --- | --- | --- |
| SNT | 3CLpro^WT^ | 0.0595 |  | 12.4 |  |
|  | 3CLpro^T21I/E166V^ | 20.6 | 346 | 14213 | 1146 |
|  | 3CLpro^L50F/E166V^ | 22.0 | 369 | 27360 | 2206 |
|  | 3CLpro^A173V^ | 0.0525 | <1 | 22.5 | 1.81 |
| NMT | 3CLpro^WT^ | 0.0547 |  | 7.80 |  |
|  | 3CLpro^T21I/E166V^ | 62.5 | 1142 | 34290 | 4396 |
|  | 3CLpro^L50F/E166V^ | 71.2 | 1301 | 47145 | 6044 |
|  | 3CLpro^A173V^ | 0.134 | 2.45 | 23.7 | 3.03 |
| BFT | 3CLpro^WT^ | 0.0310 |  | 5.10 |  |
|  | 3CLpro^T21I/E166V^ | 0.189 | 6.00 | 44.5 | 8.73 |
|  | 3CLpro^L50F/E166V^ | 0.163 | 5.20 | 58.0 | 11.4 |
|  | 3CLpro^A173V^ | 0.0299 | <1.0 | 8.07 | 1.58 |
| EST | 3CLpro^WT^ | 0.0587 |  | 8.88 |  |
|  | 3CLpro^T21I/E166V^ | 1.17 | 19.9 | 355 | 40.0 |
|  | 3CLpro^L50F/E166V^ | 2.19 | 37.3 | 1300 | 146 |
|  | 3CLpro^A173V^ | 0.0415 | <1.0 | 20.9 | 2.35 |

NMT, nirmatrelvir; SNT, simnotrelvir; BFT, bofutrelvir; EST, ensitrelvir.
